# Probing the floral developmental stages, bisexuality and sex reversions in castor (*Ricinus communis* L.)

**DOI:** 10.1038/s41598-021-81781-9

**Published:** 2021-02-19

**Authors:** Sujatha Thankeswaran Parvathy, Amala Joseph Prabakaran, Thadakamalla Jayakrishna

**Affiliations:** 1 ICAR-Indian Institute of Oilseeds Research, Rajendranagar, Hyderabad, Telangana 500030 India; 2 Present Address: ICAR-Indian Institute of Agricultural Biotechnology, Ranchi, Jharkhand 834010 India; 3 Present Address: ICAR-Sugarcane Breeding Institute, Coimbatore, Tamil Nadu India

**Keywords:** Shoot apical meristem, Plant molecular biology, Flowering

## Abstract

Castor (*Ricinus communis* L) is an ideal model species for sex mechanism studies in monoecious angiosperms, due to wide variations in sex expression. Sex reversion to monoecy in pistillate lines, along with labile sex expression, negatively influences hybrid seed purity. The study focuses on understanding the mechanisms of unisexual flower development, sex reversions and sex variations in castor, using various genotypes with distinct sex expression pattern. Male and female flowers had 8 and 12 developmental stages respectively, were morphologically similar till stage 4, with an intermediate bisexual state and were intermediate between type 1 and type 2 flowers. Pistil abortion was earlier than stamen inhibition. Sex alterations occurred at floral and inflorescence level. While sex-reversion was unidirectional towards maleness via bisexual stage, at high day temperatures (T_max_ > 38 °C), femaleness was restored with subsequent drop in temperatures. Temperature existing for 2–3 weeks during floral meristem development, influences sexuality of the flower. We report for first time that unisexuality is preceded by bisexuality in castor flowers which alters with genotype and temperature, and sex reversions as well as high sexual polymorphisms in castor are due to alterations in floral developmental pathways. Differentially expressed (male-abundant or male-specific) genes *Short chain dehydrogenase reductase 2a* (*SDR*) and *WUSCHEL* are possibly involved in sex determination of castor.

## Introduction

Plant sexual diversity forms the basis of taxonomy and has evolved to achieve mating success in flowering plants^1–6^. Sex expression or sexuality in plants can be altered in response to changes in environment or age of plant, and such lability in sex expression is significant in long term survival and adaptation of a species^7,8^.

Castor (*Ricinus communis* L.) is an industrially important oilseed crop, the non-edible oil mainly used as a lubricant due to high viscosity^9^. The inflorescence of castor is monoecious, monoclinal, primarily wind-pollinated raceme, bearing unisexual flowers, with female flowers found on the apex and male flowers borne at the bottom of inflorescence^10^. Though a diploid (2n = 20), belonging to a monotypic genus of family *Euphorbiacea*, castor exhibits racial differences for sex tendency^11^. High polymorphism of sex expression in castor ranges from genotypes having spikes which are completely pistillate to completely staminate, with interspersed staminate flowers or ISF (male flowers interspersed throughout in the pistillate spike after capsule formation), monoecious with apical ISF (male flowers interspersed in the apical region of spike), or apically non-interspersed, or with terminal hermaphrodite flower and having monoecious sex variants showing variation in percentage as well as the relative position of male and female flowers within the genotype^10,12,13^. In addition, the highly unstable pistillate character reverts to monoecism^14^. The hereditary instability affecting sex, manifested as sex reversal in pistillate lines is a widely prevalent phenomenon in castor, which negatively influences hybrid seed purity. Such reversions may be early or late, when it occurs respectively at lower or higher than quaternary orders of spikes, later reversions resulting in more females in the progenies^10,15^. During hybrid seed production, the reverted pistillate lines themselves act as a source of pollen. Roguing of reverted spikes is cumbersome and increases the production costs. Conventional method of hybrid seed production retains 20–25% monoecists for maintenance of pistillate lines, resulting in high percentage of monoecists and early revertants in seed production plots. In a modified method for maintenance of pistillate lines, interspersed staminate flowers are induced during summer and hybrid seed production is carried out during rabi season^16^. This method requires less roguing of sex-revertant spikes, but is highly season-dependent.

Sex determination and reversal in plants are poorly investigated issues of developmental biology, inspite of their theoretical and practical significance^17^. Sex expression in castor may be controlled by genetic, cytogenetic, epigenetic, physiological and developmental factors. Analysis of the genetic factors influencing sexual polymorphism in castor has indicated three different pistillate mechanisms viz., N-type, governed by a recessive sex-switching gene, S-type derived from sex reversals and NES type, homozygous for N-pistillate gene and with environmentally sensitive genes for ISF^10,18–21^. Interspersed sexuality resulted from a combination of hereditary factors for femaleness and ISF*,* where some genes for ISF (*id*) were environmentally sensitive, determined by a system of polygenes and others were environmentally resistant^10,13,14,20^. High temperature was found to cause maleness and NES type contributed to instability^22^. Environmental and intrinsic factors such as season, temperature, photoperiod, light, age of plant, nutrition level, vegetative activity, pruning and plant hormones affect sex expression in castor^11,14,20,23–29^. Though pachytene chromosome morphology did not vary between monoecious and pistillate lines, haploid plants had sterile racemes and trisomic castor plants had only male flowers, with occasional single terminal hermaphrodite flowers, indicating that cytogenetic or chromosomal aberrations may influence sex expression^12,30,31^. More than 3000 differentially expressed genes were identified between monoecious and pistillate genes, possibly involved in sex determination in castor^29^.

Though several mechanisms have been postulated to govern sex lability in castor, the reproductive biology and evolution of monoecy is not explained in the context of the complex and intriguing phenomenon of sex expression. Sex determination systems in plants have evolved from hermaphroditic ancestors^32,33^. Bisexual flowers are ancestral and unisexual flowers have evolved many times independently^34^. Understanding the molecular mechanisms of unisexuality has been a long-standing quest in plant biology, though several developmental and genetic mechanisms underlying unisexual flower development are predicted^35,36^.The study of organogenesis of unisexual flower in *Euphorbiaceae* is extremely scarce, even for the most common and best-known species, such as castor (*Ricinus communis* L.)^37^.

The present study aims to understand the nature and causes such as developmental, environmental and molecular mechanisms of sex reversion and sex variability in castor. The reproductive biology, inflorescence development and architecture, male and female flower developmental pathways, effect of temperature on sex expression and gene expression in various tissues were studied using different castor genotypes with distinct sex expression phenotypes, so that sex expression can be controlled in a desirable manner by physical, chemical, physiological or biotechnological means.

## Results

### Inflorescence development has distinct stages and inflorescence architecture varies with genotypes

**Figure 1:**
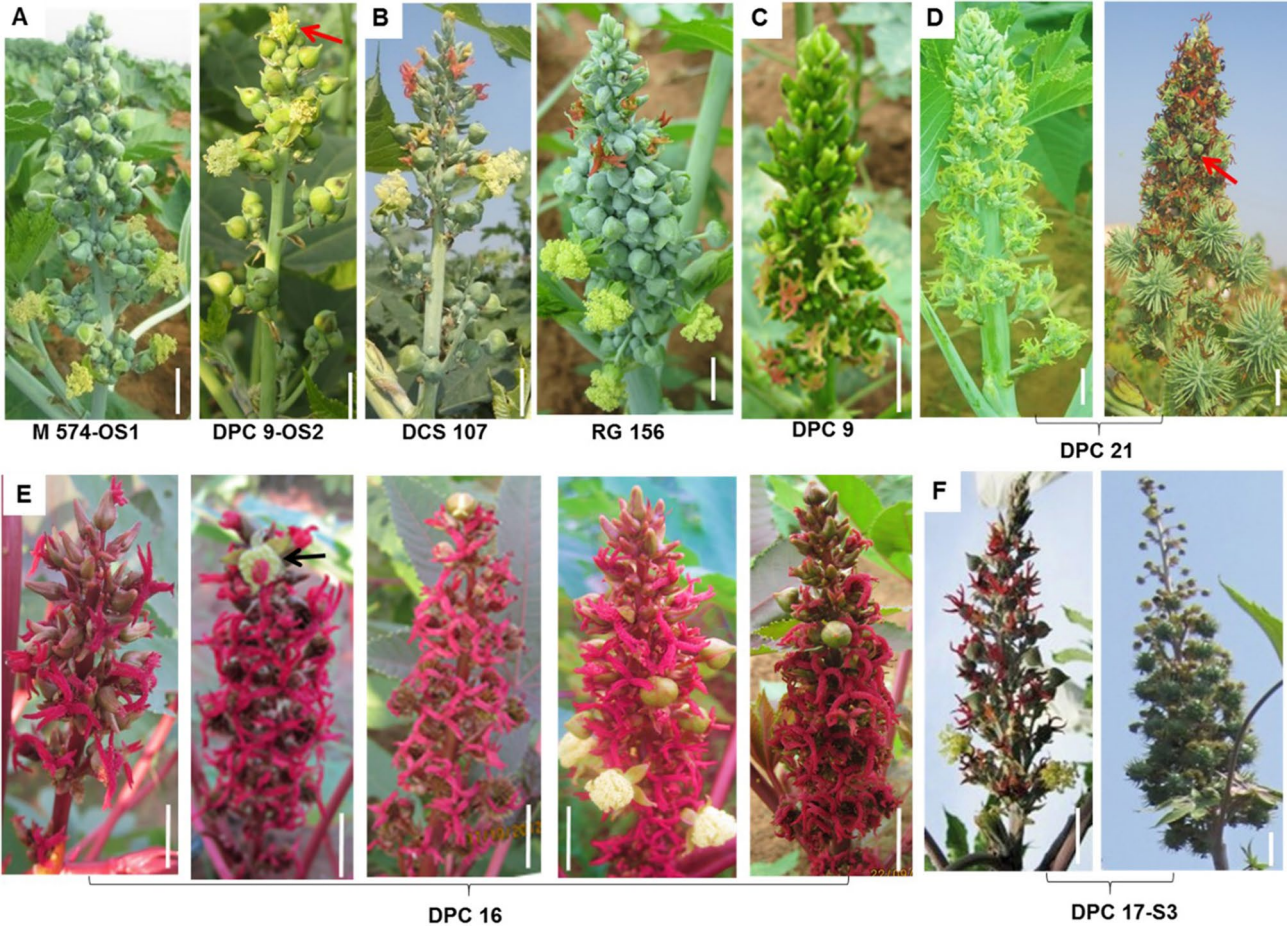
Sex variability in castor genotypes. Inflorescences which are (**A**) Predominantly male (above quarternary branching orders) with terminal bisexual flower. (**B**) Monoecious with 30–50% male flowers (DCS 107) and 70–80% male flowers (RG 156) (**C**) Completely pistillate. (**D**) Interspersed staminate flowers (ISF) at capsule maturation. (**E**) Completely pistillate predominantly pistillate with single terminal hermaphrodite flower, few hermaphrodite flowers, male flowers at random positions and terminal female or male flower and (**F**) Apical ISF. Genotype names are indicated below. Scale bar: 1 cm.

Castor exhibits determinate growth, where main stem and subsequent branches terminate in a racemose monoecious inflorescence, but diverse sex patterns were observed. Different castor genotypes with distinct and diverse sex phenotypes ranging from completely pistillate to completely staminate and monoecious with different proportion and position of male and female flowers were used in the present study (Fig. 1).

**Figure 2:**
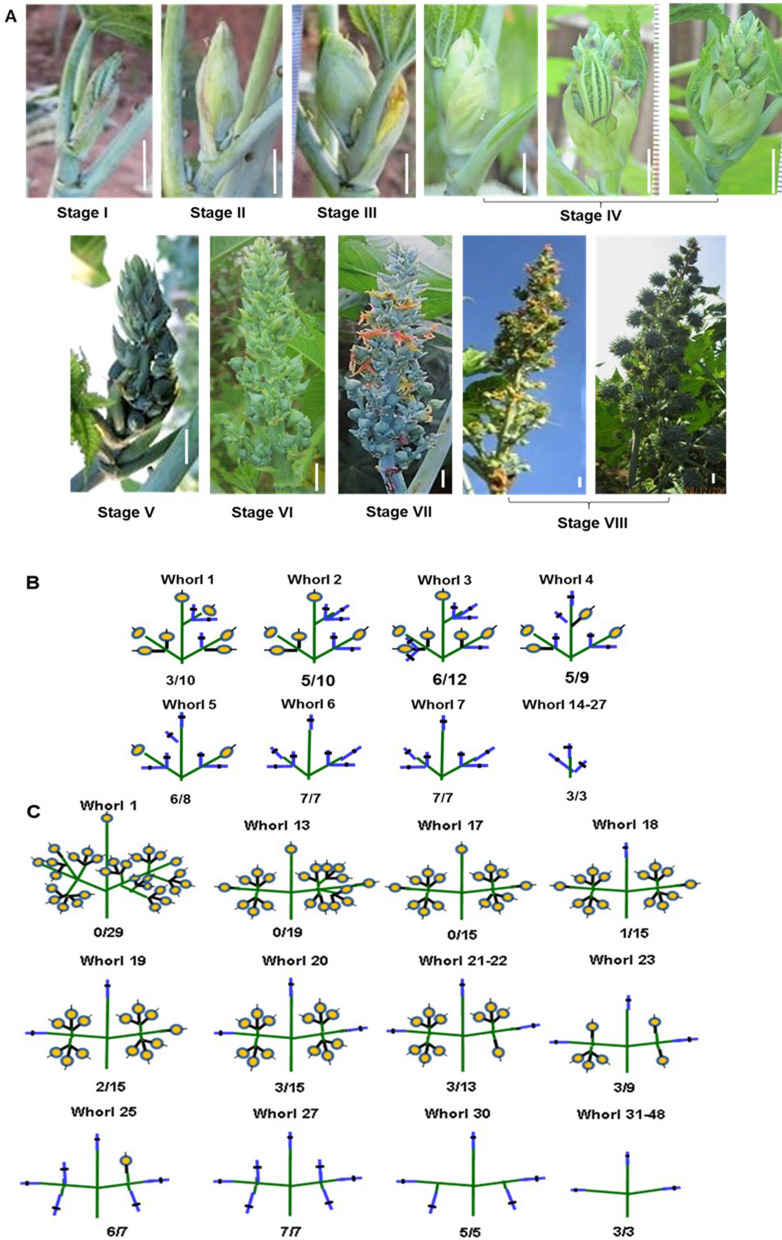
Morphological developmental stages and architecture of inflorescence. (**A**) Developmental stages of inflorescence (stage I-VIII) from primordia initiation to capsule setting. (DCS 107). (**B,C**) Inflorescence architecture in monoecious genotypes DCS 107 (**B**) and RG 156 (**C**). Floral whorl numbers counted from bottom of inflorescence and proportion of female flowers (fraction) in a whorl are shown. Quantitative information on inforescence growth, architecture and time for transtion to each stage are provided in Supplementary Figs. S2–S5 and Supplementary Tables S2 and S3.

The shoot apical meristem (SAM) was enveloped by many whorls of bracts and leaves and not visible externally. During vegetative growth, the dome-shaped shoot apical meristem produced leaf primordia. Each leaf at a node was subtended by a bract. The vegetative stage terminated with initiation of inflorescence primordium. Based on ontogeny of distinct morphological events, eight morphological stages of inflorescence development, from floral initiation to capsule setting, were identified in castor (Fig. 2A, Supplementary Table S1). At stage I, inflorescence primordium initiated, but was not distinct from vegetative shoot apex. At stage II, inflorescence primordium was externally visible as a bulge. The inflorescence increased in girth and had fully formed flower buds at stage III. Stage IV represented spike opening from tip downwards, with three sub-stages to mark beginning, half and three-quarter opened inflorescence. Increase in girth of inflorescence from stage I to stage IV are shown (Supplementary Fig. S1). Spike emerged fully at stage V and elongated at stage VI. During elongation, flower buds were added to floral whorls. Anthesis or flower bud opening (stage VII) and capsule formation (after pollination) and maturation (stage VIII) occurred. Spike elongation continued during capsule maturation. For scoring the sex phenotype, end of stage VI, just before anthesis was ideal for most genotypes, since elongation and addition of the floral whorls would mostly be complete. Stage VIII was scored for ISF.

Time taken for transitions to each stage and anthesis pattern varied with the genotype (Supplementary Fig. S2; Supplementary Tables S2A and B). In monoecious DCS 107, a distinct stage of elongation (stage VI) existed before anthesis, but in other genotypes elongation was distinct during or after anthesis. In DPC 9, anthesis started immediately after emergence (3 days), when compared to other genotypes (7–10 days). Elongation was greater during capsule formation and maturation (Supplementary Fig S2; Supplementary Tables S2C and D). Studies on anthesis pattern revealed that in monoecious DCS 107, anthesis first occurred in the lower whorl of female flowers, but in monoecious RG 156, 1–2 male flowers opened first, followed by lower whorls of female flowers and majority of male flowers remained unopened even during capsule formation. In pistillate DPC 9 and DPC 21, bottom whorl of female flower buds opened first and continued up. In DPC 17-S3 (monoecious apical ISF), the bottom whorls of female flowers opened first followed by middle whorl. The male flowers at the bottom of the inflorescence opened only after anthesis of 75% of female flowers and mostly during capsule formation stage.

The inflorescence architecture in terms of number of floral whorls, sex and relative position of flower buds varied in different genotypes (Supplementary Fig. S3). Bottom whorls were more branched and with more number of buds than top whorls. In monoecious genotypes (DCS 107 and RG 156) proportion of male flowers gradually decreased, while that of female buds gradually increased to completely female whorls towards top. Monoecious genotypes also differed in inflorescence architecture. In DCS 107, the female flower bud appeared with in first to third whorl from bottom (Fig. 2B), whereas in RG 156, not a single female bud was observed in lower whorls (Fig. 2C). In pistillate lines DPC 9 and DPC 21, female buds alone were seen in all the whorls, although male flowers were observed during sex reversion or ISF in DPC 9 and capsule formation (ISF) in DPC 21. In M 574-OS1, only male flowers were observed from quaternary orders but the terminal flower was occasionally bisexual in some spikes. In DPC 17-S3 (with apical ISF spike), male flowers were found throughout the inflorescence, but completely female whorls occurred occasionally in between (Supplementary Table S3). In top whorls with triplet buds, one of the two lateral buds were male and the terminal bud was mostly female. Also, few slightly pointed male flowers towards the spike tip were observed in DPC 17-S3 and DCS 107, while some female flowers of RG 156 and DPC 9 were round than elongated (Supplementary Fig. S4), which were more prominent during summer. For a given length, the number of floral whorls decreased with branch order (tertiaries had less whorls than secondaries). Every spike order in a genotype had definite number of floral whorls during emergence which did not increase significantly later, but increase in floral whorls during inflorescence elongation and development was noticed in monoecious DCS 107 and staminate M 574-OS1 (p < 0.05) (Supplementary Table S3; Supplementary Fig. S5) indicating potential of sex lability with environmental conditions during growing season in these genotypes. The number of flowers in a floral whorl (especially in lower whorls) may increase, due to continuous development of flower primordia in triplets, resulting in more branching.

### Apical meristem shows chronology of organ developmental stages during inflorescence initiation and development

Inflorescence primordia initiation at stage I, though not distinct externally and inflorescence development at stage II were identified by histology sections and SEM. The much-condensed internodes near apical meristem, enveloped by bracts were revealed only after emergence of each leaf at a node, which takes approximately 3–4 days. Inflorescence primordia initation usually occurs at 8-12 nodes in the primary branch or later depending on genotype, growth conditions and seed quality (data not shown). Fully emerged inflorescence (stage V) appeared 3–5 nodes later than or 2–3 weeks after inflorescence initiation at stage I.

**Figure 3:**
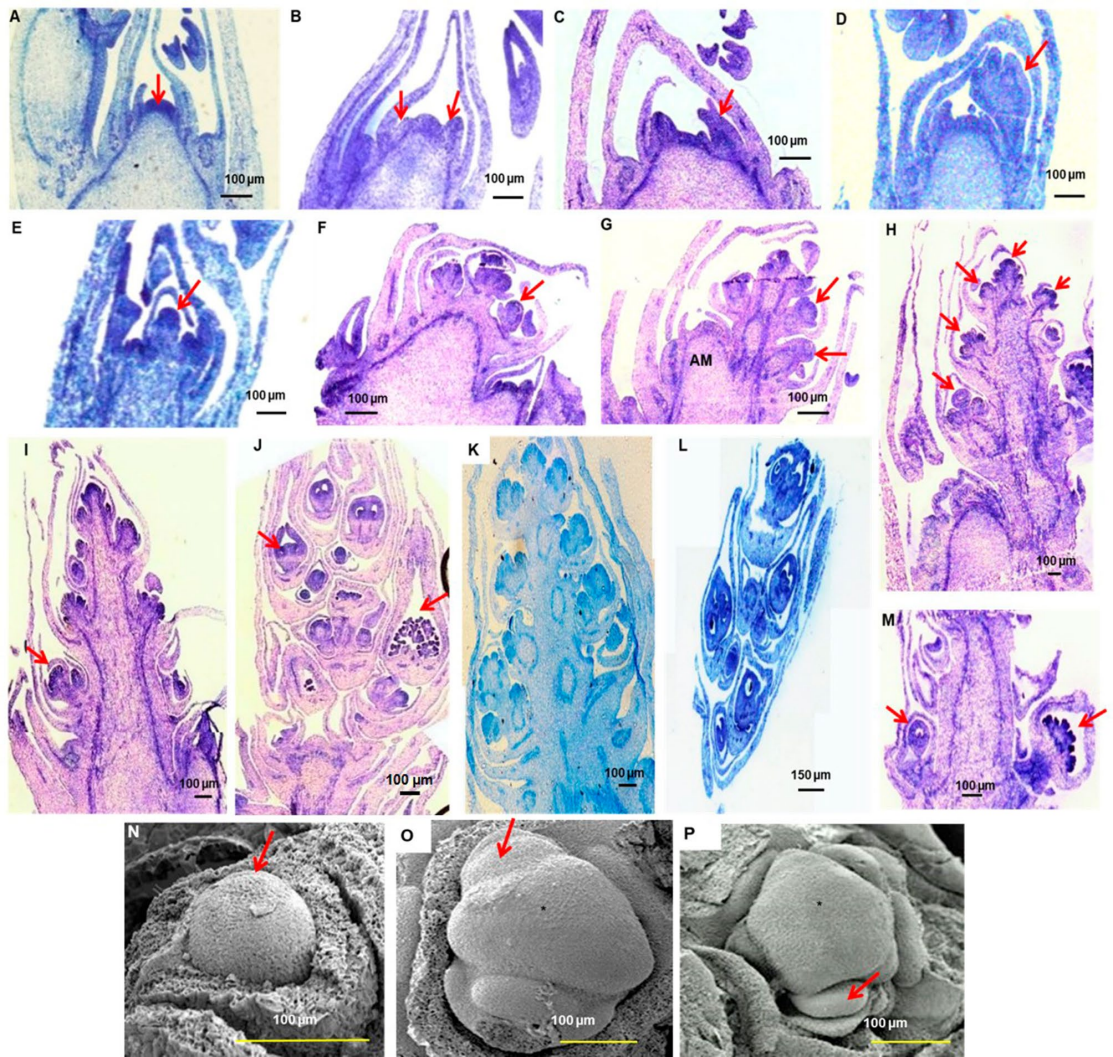
Chronology of development stages of apical meristem during raceme formation. (**A–D**) Vegetative shoot apical meristem (SAM) at different developmental stages. Arrows indicate SAM and bract or leaf primordia (**E**) Inflorescence primordia initiation. (**F,G**) Floral primordia differentiation. AM is apical meristem. Arrows indicate floral meristem (**H–J**) Inflorescence elongation and differentiation of buds. Arrows indicate male and female bud (**K–M**) Lower floral whorls with (**K**) only male (RG 156) (**L**) only female (DPC 9) and (**M**) both male and female flowers (DCS 107). Sections are of ×40 magnification. (**N–P**) Scanning Electron Micrographs of shoot apical meristem (SAM) (**N**) SAM during vegetative growth (arrow) (**O**) Bract and/or leaf primordia (arrow). Asterisk indicates SAM. (**P**) SAM during inflorescence initiation. Arrow indicates floral meristem. Asterisk indicates inflorescence meristem. Scale bars are shown below.

During the vegetative phase, the dome-shaped apical meristem enveloped within bracts had 2-to-3 protuberances (Fig. 3A,B), which later differentiated into leaf-like structures that emerged disrupting the bracts (Fig. 3C,D). The protrusion can be also the bract. The inflorescence primordia initiation was marked by elongation of apical meristem beyond the youngest leaf primordium, to become the main axis of inflorescence (Fig. 3E), followed by floral primordial differentiation, where lateral regions of meristematic activity or individual floral buds were differentiated on the axis (Figs. 3F,G). Growth and elongation of inflorescence axis occurred in a centripetal fashion from bottom to top, adding new floral whorls as it elongated (Fig. 3H). Meristematic growth continued at inflorescence tip while the lateral regions continued differentiating (Fig. 3I). The buds in the lower whorls matured first and individual flower buds were at different developmental stages (Fig. 3J). In monoecious genotypes, male buds differentiated first and female buds were observed after the inflorescence elongated further by 2 nodes. In RG 156, male flowers alone were observed, in the lower whorls (Fig. 3K), while in pistillate line DPC 9, female flower buds alone were observed at different developmental stages (Fig. 3L). In DCS 107, both male and female buds differentiated (Fig. 3M). Scanning electron microscopy of the apical meristem revealed the transition from vegetative to reproductive stage, marked by several protrusions of bracts and individual flower bud primordia beneath the apical meristem (Figs. 3N–P).

### Unisexual flowers of castor have an intermediate bisexual stage

**Figure 4:**
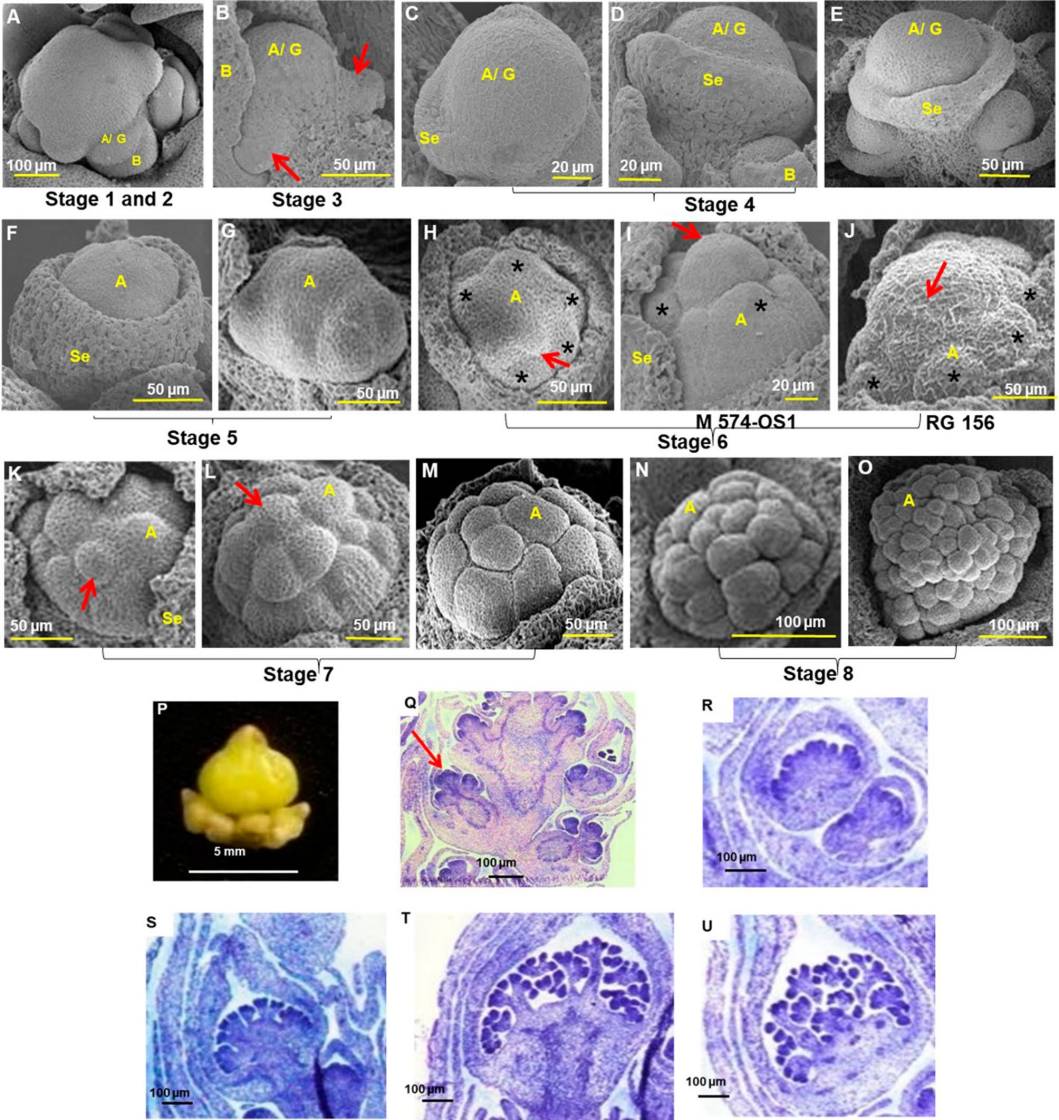
Stages of male flower development. (**A**) Bract (stage 1) and floral primordia (stage 2) initiation. (**B**). Division in central floral primordia (stage 3). Arrows indicate lateral floral primordia. (**C–E**) Sepal primordia initiation (stage 4). (**F,G**) stage 5 with (**F**) cup shaped flower bud and (**G**) irregular floral meristem. (**H–J**) Stage 6 with 6–7 celled structure in (**I**) M574-OS1 (**J**) RG 156. Arrow indicates central cell and asterisk the peripheral cells. (**K–M**) Growth arrest of pistil primordium (stage 7). (**N,O**) Stamen development (stage 8). (**P**) Male flowers in triplets. (**Q–U**) Histological sections showing development of male flower bud (×40 magnification). Scale bars are shown below. *A* androecium, *B* bract, *G* gynoecium, *Se* sepal**.**

In male flowers, eight developmental stages were identified. From apical meristem of inflorescence, a lateral flap-like outgrowth (bract primordium), emerged at stage 1 which expanded with growth of inflorescence meristem. At stage 2, a dome-like cushiony growth (floral meristem) occurred beneath the inflorescence meristem, near centre of primordial bract (Fig. 4A). The floral meristem covered by a bract divided to form two lateral floral primordia at stage 3 (Fig. 4B). At stage 4, sepal primordia initiated from the periphery of central floral meristem to form a saucer-shaped structure (Fig. 4C,D). Castor flowers do not have petals. Each floral whorl with triplet flower buds were at different levels of development, the central bud being more mature than lateral buds (Fig. 4E). Both male and female flowers were morphologically indistinguishable (saucer-shaped) and had common developmental stages till stage 4, the developmental programmes being distinct from stage 5.

At stage 5 of male flower development, the sepals grew partially covering the central floral meristem, forming a cup-shaped structure, while surface of central floral meristem appeared irregular due to divisions (Fig. 4F,G). At stage 6, central floral meristem completely covered by sepals, divided to 6-7 cells, with a distinct central cell (Fig. 4H). This was the bisexual state of male flower. In M 574-OS1 the central cell was prominent, dome-like raised structure when compared to the surrounding peripheral cells (Fig. 4I), but in RG 156 (monoecious), the central cell was not distinct, though polarity into central and peripheral cells was observed (Fig. 4J). At stage 7, the central cell lost its polarity, aborted without developing further and flattened out after random multiple divisions (Fig. 4K–M), indicating that the central cell may be pistil primordium which was arrested in male flowers. A series of divisions occurred in floral meristem at stage 8, to form stamens with anthers and filaments (Fig. 4N,O).

The flower buds of castor developed in triplets, (Fig. 4P,Q), during early developmental stages, when central floral meristem divides to two lateral floral meristems, which continue further divisions, resulting in a floral whorl. Stamens having globular anthers at the tip of short filaments, developed from the central meristematic mass of cells (Fig. 4R,S). Further divisions increased the number of bilocular anthers and the filaments elongated with growth of the flower bud (Fig. 4T,U).

**Figure 5:**
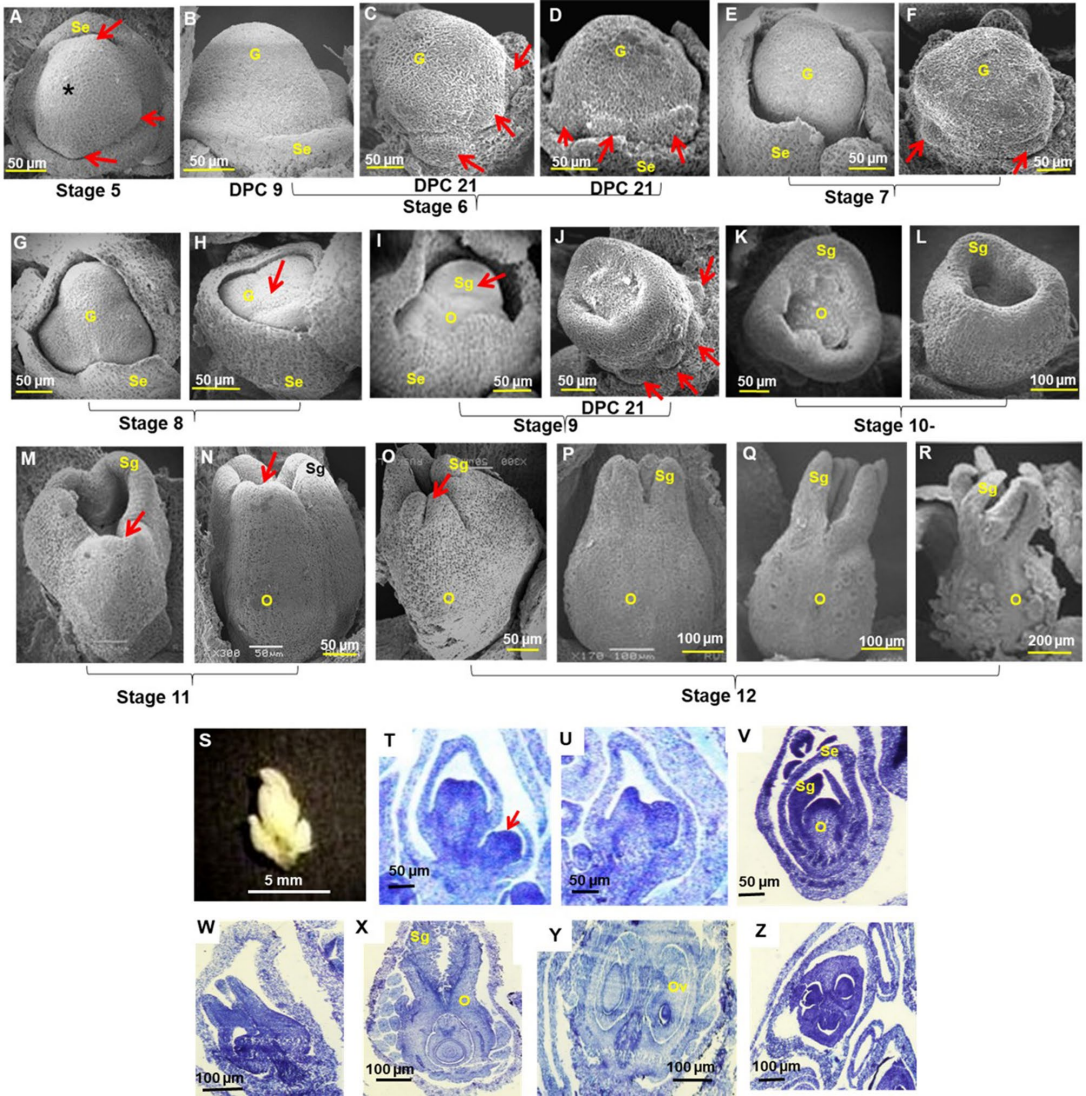
Stages of female flower development. Stages 1–4 are similar to male flowers (**A**) Stage 5 with stamen primordia (arrows). Asterisk indicates floral meristem. (**B–D**) Growth of central floral meristem (stage 6). (**E,F**) Cup-shaped gynoecium (stage 7). (**G,H**) Triangular gynoecium with central circular ovary (arrows) at stage 8. (**I,J**) Development of stigma primordia (arrow) at stage 9. Stamen primordia (arrows) are prominent in ISF line DPC 21. (**K,L**). Growth of stigma (stage 10) (**M,N**) Bifid stigma formation (stage 11). (**O–R**) Ovary and stigma development (stage 12). Stamen primordia (arrows) are distinct in ISF line DPC 21 from stages 6–9. (**S**) Female flowers in triplet. (**T–Z**) Histological sections showing stigma and ovule development. Scale bars are shown below. Sections are of ×40 magnification. *B* bract, *G* gynoecium, *O* ovary, *Se* sepal, *Sg* stigma.

In female flowers, 12 developmental stages were identified, their development similar to male flowers till stage 4. At stage 5, some lateral protrusions (stamen primordia) were observed at the periphery of floral meristem, inner to the sepal primordia (Fig. 5A). At stage 6, when the central tissue grew and elongated vertically, these stamen primordia were not distinct in pistillate DPC 9 (Fig. 5B), but were distinct in ISF line, DPC-21 (Fig. 5C,D). At stage 7, central protuberance (pistil primordia) was near-triangular with flat surface and partially covered by calyx to form a cup-shaped structure (Fig. 5E,F). At stage 8, the three-sided central tissue (ovary) became distinct, with circular central region flanked by three peripheral growths (primordia of stigma) from edges of ovary (Fig. 5G,H). Stigma developed as wing-like outgrowths from the edges of ovary at stage 9 (Fig. 5I). The stamen primordia beneath the ovary was more conspicuous in ISF line (Fig. 5J). Sepals covered the flower bud and further development of gynoecium occurred within calyx. At stage 10, the peripheral structures (stigma) outgrew the central ovary to form three distinct leaf-like structures (Fig. 5K,L). Three grooves formed in each stigma, initiating bifid stigma development, at stage 11 (Fig. 5M,N). At stage 12, the stigma elongated to form six finger-like structures representing three bifid stigma and ovary developed fully with spiny outgrowths (Fig. 5O–R). Calyx elongated with growth of gynoecium inside and the fully developed female flower bud was pointed.

Female flower buds also developed in triplets (Fig. 5S,T). Stigma development from central ovary (Fig. 5U,V) and development of ovules inside ovary were clear in histological sections (Fig. 5W,X). A single ovule developed from each of the 3 cavities formed in a vertical plane inside ovary (Fig. 5Y,Z). The flowers were trimerous with three sepals, three-loculed ovary and three-lobed bifid stigma.

### Bisexuality and reversion to bisexuality occur in castor flowers

**Figure 6:**
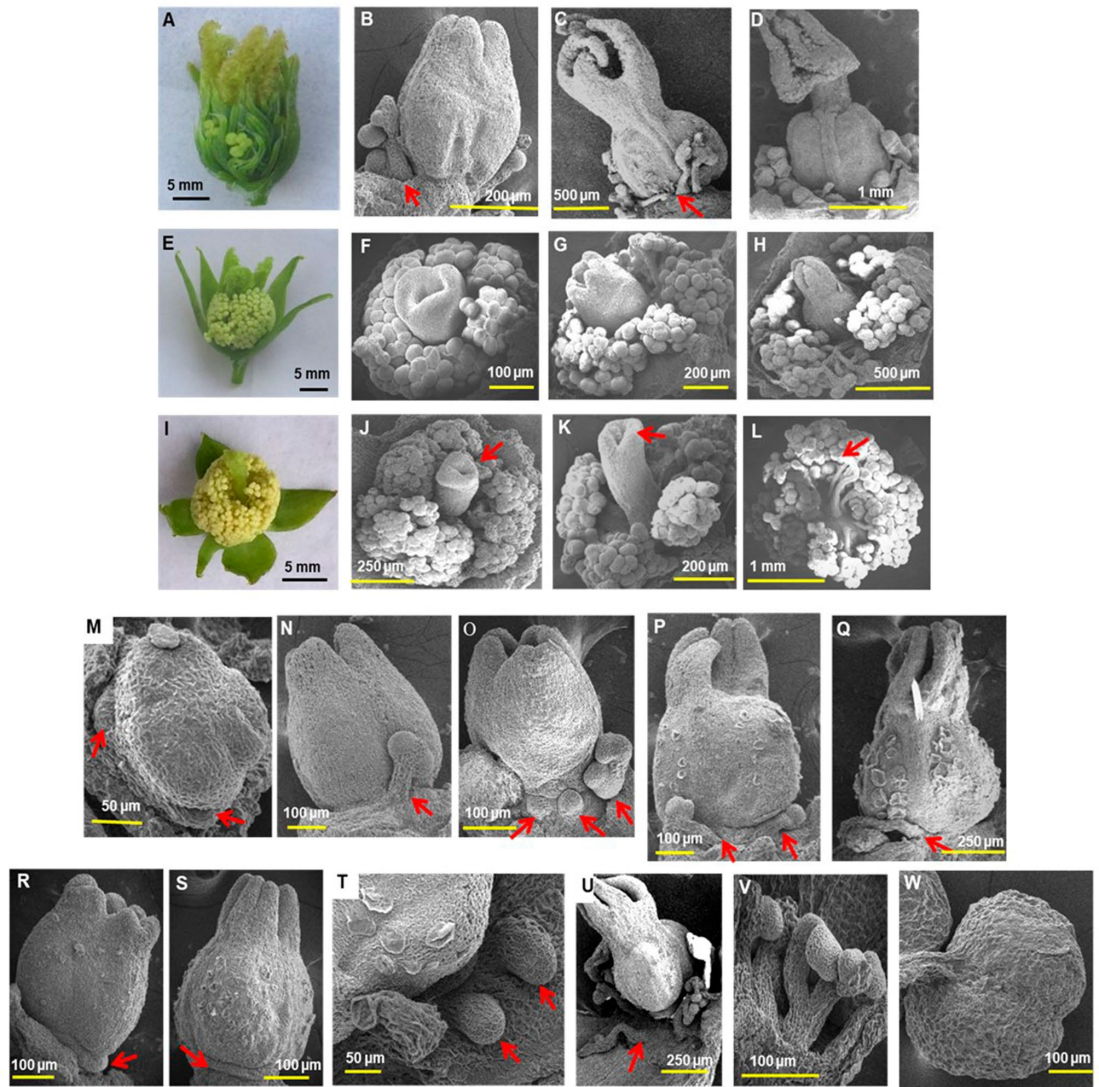
Bisexual flowers and reversion to bisexuality in castor. Bisexual flowers with (**A–D**) Rudimentary stamens (**E–H**) Equally developed carpel and stamens and (**I–L**) Rudimentary pistil. (**M–Q**) Reversion to bisexuality in female flowers at (**M**) stage 6 (**N,O**) stage 11 and (**P,Q**) stage 12. **(R–U)** Origin of rudimentary stamens. (**V**) Section of flower showing origin of rudimentary stamens. (**W**) Larger anthers in rudimentary stamens. Arrows indicate stamen primordia or rudimentary stamens (**B,C,M–V**) or rudimentary pistils (**J–L**). Scale bars are shown below.

Occurrence of bisexual flowers having fully developed male and female organs was rare and bisexual flowers did not occur in all castor genotypes under normal conditions. Spikes of DPC 16, DPC 9-OS2 and M 574-OS1 had terminal hermaphrodite flower. In DPC 16 and DPC 9-OS2, few bisexual flowers were also found near the spike tip. A gradation in bisexuality exists in castor, where three categories of bisexual flowers were identified such as, bisexual flower which was predominantly female, having well-developed ovary and few rudimentary stamens (Fig. 6A–D), bisexual flower with equally and fully developed gynoecium and androecium (Fig. 6E–H) and predominantly male bisexual flower with underdeveloped tubular ovary, rudimentary stigma or without bifid stigma (Fig. 6I–L). The terminal bisexual flowers generally had equal development of male and female structures.

Out-crossing in pistillate line DPC 9 (DPC 9-OS2) resulted in monoecious inflorescence with bisexual flower(s) at and near tip. In lower branch orders, bisexual flowers had equally developed ovary and stamens, but transited to predominantly male bisexual flower, with rudimentary tubular ovary, and gradually to completely male flower. The terminal bisexual flower progressively became male and had rudimentary gynoecium during stages of transition, the progressive transition being noticed in bisexual flowers of different spikes of higher order and not of the same spike. Similar sex expression was observed in M 574-OS1 and in few plants of VP1 (data not shown).

At high day temperatures during summer, sex reversion was unidirectional towards maleness, at floral and inflorescence level, from female to bisexual, in female flowers, and from bisexual to male, in spikes with terminal bisexual flowers. Female flowers of both monoecious and pistillate lines reverted to bisexual flowers with rudimentary stamens, at most developmental stages from stages 6 to 12 (Fig. 6M–Q). The aborted stamen primordia, which remained dormant as a layer of cells beneath the ovary (Fig. 6R,S), resumed development as small protrusions or bulges at the base of the ovary during reversion (Fig. 6T), to result in rudimentary stamens. Such reversions occurred even after complete pistil development, but simultaneous development of gynoecium and androecium were not observed in reverted female flowers. When reversion occurred during later stages of female flower development (after complete development of gynoecium), rudimentary stamens appeared without complete development, with less branching and development which was limited to filament bifurcation (Fig. 6U,V). Also, anthers in reverted stamens of bisexual flowers of DPC 9-OS2 were larger in size (200–300 µm diameter) when compared to normal bi-lobed anthers (30–50 µm in diameter), in immature or mature male flower buds of size nearly 200 µm and 400 or 800 µm diameter respectively, suggesting failure or reduced number of divisions in the central androecium tissue (Fig. 6W). Thus, at high temperatures, sex reversion towards maleness was observed as transition from female to bisexual in same flower and from bisexual to male in flowers of different spikes.

### Extrinsic factors like temperature play a major role in sex determination in castor

**Figure 7:**
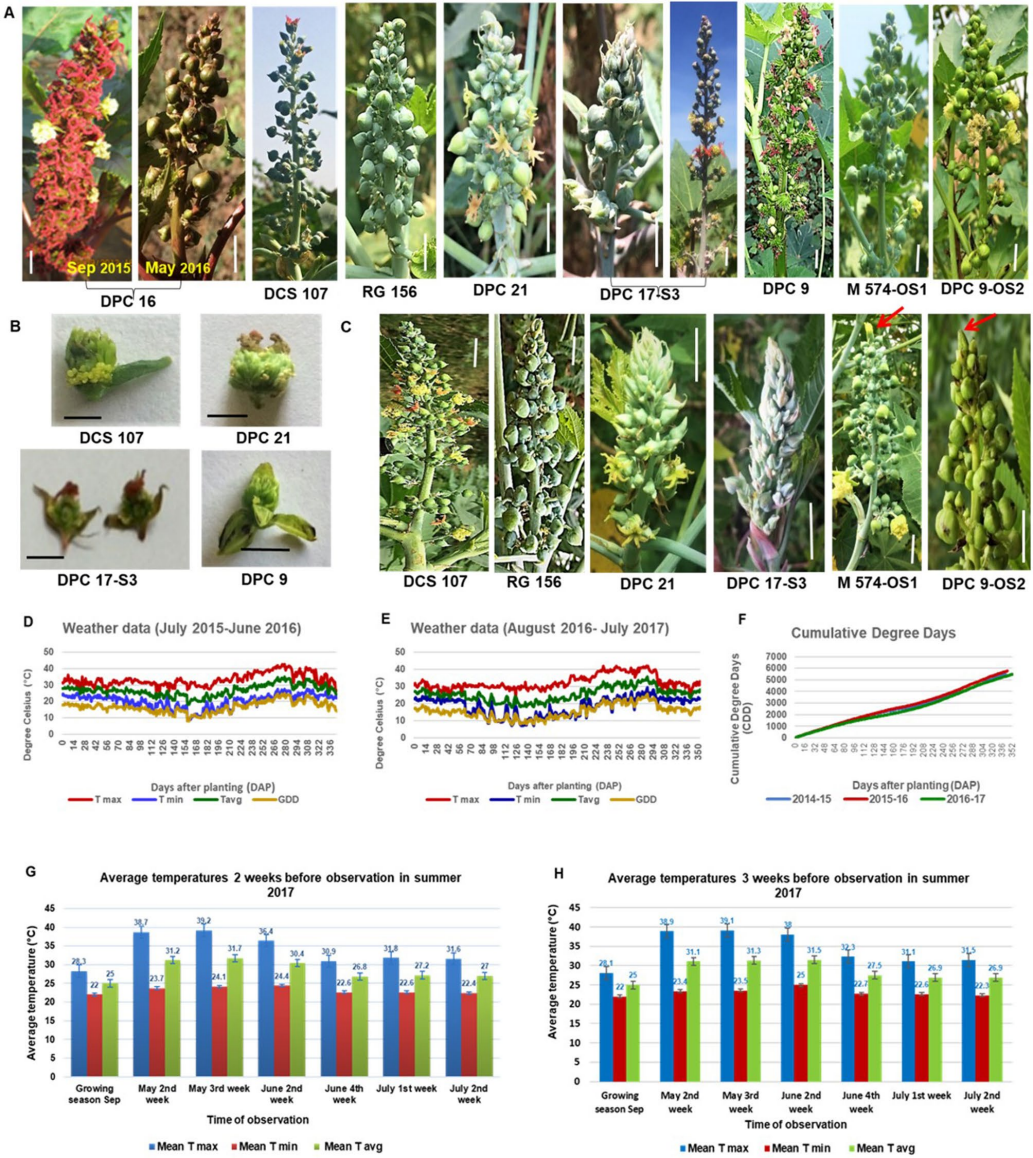
Sex expression in castor in response to temperature. (**A**) Alteration of sex phenotypes towards completely or predominantly male spikes in castor genotypes at high temperatures during summer in March -May (T_max_ 39–42 °C). (**B**) Bisexual flowers in castor genotypes in summer (**C**) Restoration of normal phenotype in June-July at normal temperatures (T_max_ 28–33 °C). Genotype names are indicated below. Arrows indicate terminal female flower. Weather data during growing seasons (**D**) 2015–16 and (**E**) 2016–17. (**F**) Cumulative degree days during 3 growing seasons 2014–15, 2015–16 and 2016–17. Temperature parameters (mean of T_max_, T_min_ and T_avg_) existing for (**G**) 2 weeks and (**F**) 3 weeks before observation of altered sex phenotype in inflorescences during summer. Scale bars: 1 cm are shown.

At high temperatures in summer, though inflorescence initiation and development were inhibited, new flushes emerged in 8–9 months old crop, when irrigated. In summer season (March- May, 2016 and 2017 with maximum day temperatures or T_max_ of 39–42 °C), the sex of inflorescences altered towards maleness in most genotypes, even in varieties and germplasm accessions with stable sex expression over several generations. In summer, the spikes were shorter, with widely spaced floral whorls, less number of buds per floral whorl, sparsely distributed buds with reduced size, increased proportion of male flowers and with bisexual flowers (Fig. 7A). In DPC 16, instead of predominantly pistillate spikes observed in September 2015, predominantly staminate inflorescence with larger male flowers and very few female flowers, were observed in May 2016 (Fig. 7A). Male flowers were interspersed throughout the inflorescence (ISF) in monoecious DCS 107, while in RG 156, proportion of male flowers was increased to 90–95%, terminal flower was male instead of female and female flowers nearly round in summer. Whole spike became staminate with very few or no female flowers in DPC 21 and DPC 17-S3. ISF was observed in stable pistillate line DPC 9. Few female flowers of DPC 9 were less tapering and slightly round than normal (Supplementary Fig. S4) and had rudimentary stamens. In M574- OS1 and DPC 9- OS2, spikes were completely staminate and tip flower male. Bisexual flowers were observed seen in few plants of DCS 107 (terminal), DPC 21, and DPC 17-S3 (terminal) and DPC 9 (reverted) (Fig. 7B). Sex reversions in pistillate lines resulted in monoecious racemes, but a monoecious raceme never reverted to a pistillate raceme.

When temperatures dropped down to 28–33 °C (T_max_) by June 4^th^ week , from 38–41 °C (T_max_) during May 3^rd^ week of 2017, normal phenotype was restored in all genotypes (Fig. 7C). In M 574-OS1 and DPC 9-OS2, female flowers started appearing in terminal position in inflorescences at later orders with drop in temperature in June. The sex of the terminal flower in different inflorescences of M 574-OS1 and DPC 9-OS2 varied from female to bisexual to male with increase in temperatures during summer, and then from male to bisexual to completely female, when the temperatures dropped down after summer. This indicated that the transition of monoecious inflorescence with 20% female flowers and tip bisexual flower, to staminate inflorescence with bisexual flower and later to completely staminate inflorescence (from 4th or higher branching order onwards), was due to temperature, rather than the higher branch order or ageing that occurred during the advancement of growing season.

Weather parameters such as daily maximum temperatures (T_max_), growing degree days (GDD), daily minimum temperatures (T_min_) and daily average temperatures (T_avg_) for growing season of 2015–16 and 2016–17 showed similar patterns of change (Fig. 7D,E; Supplementary Fig S6). Cumulative degree days (CDD) calculated from date of sowing or Days After Planting (DAP) were higher for the year 2015–16 than for years 2014–15 and 2016–17 (Fig. 7F). Sex alterations in most castor genotypes were observed during mid-March to mid-June during 2015, 2016 and 2017, when most of the weather parameters GDD, T_max,_ T_min,_ and T_avg_ exhibited maximum values. The temperature conditions prevailing during inflorescence bud initiation affect the sex phenotype and the proportion of male and female flowers in the inflorescence. Hence the mean temperatures of T_max_, T_min_ and T_avg_ existing for 2–3 weeks prior to actual observation of the altered phenotype in summer were calculated, and the altered phenotypes were observed at T_max_ > 38 °C, T_min_ > 23 °C and T_avg_ > 30 °C (Fig. 7G,H). Alteration of sex phenotypes during summer towards maleness occurred in all spike orders, irrespective of the order of branching (Supplementary Table S4A). Bisexual flowers occurred in spikes of monoecious and pistillate genotypes in summer, while bisexuality and pistillateness returned in spikes of staminate genotypes with drop in temperature after summer (Supplementary Table S4B-D).

Sudden variations (rise or drop) in temperatures, rather than the absolute value of T_max_ seem to alter sex expression. In addition to temperature, genetic factors also contribute to sex variations, since completely male inflorescences were observed even in the primaries of DPC 9-OS2 during normal temperatures of growing season by August-September 2017 (Data not shown).

The mean of weather parameters existing 2 weeks before sample collection for scanning electron microscopy (Supplementary Table S5) shows that, though the DPC 21 (ISF line) samples were collected during January–February (T_max_: 28–29 °C and CDD: 2650–2856), female flowers of DPC 21 exhibited distinct stamen primordia than that of DPC 9 and RG 156 which were collected during summer (April 2016, CDD = 4032), indicating that the conspicuousness of arrested organs depends on the genotype as well.

### Differentially expressed genes may determine sexuality in castor flowers

**Figure 8:**
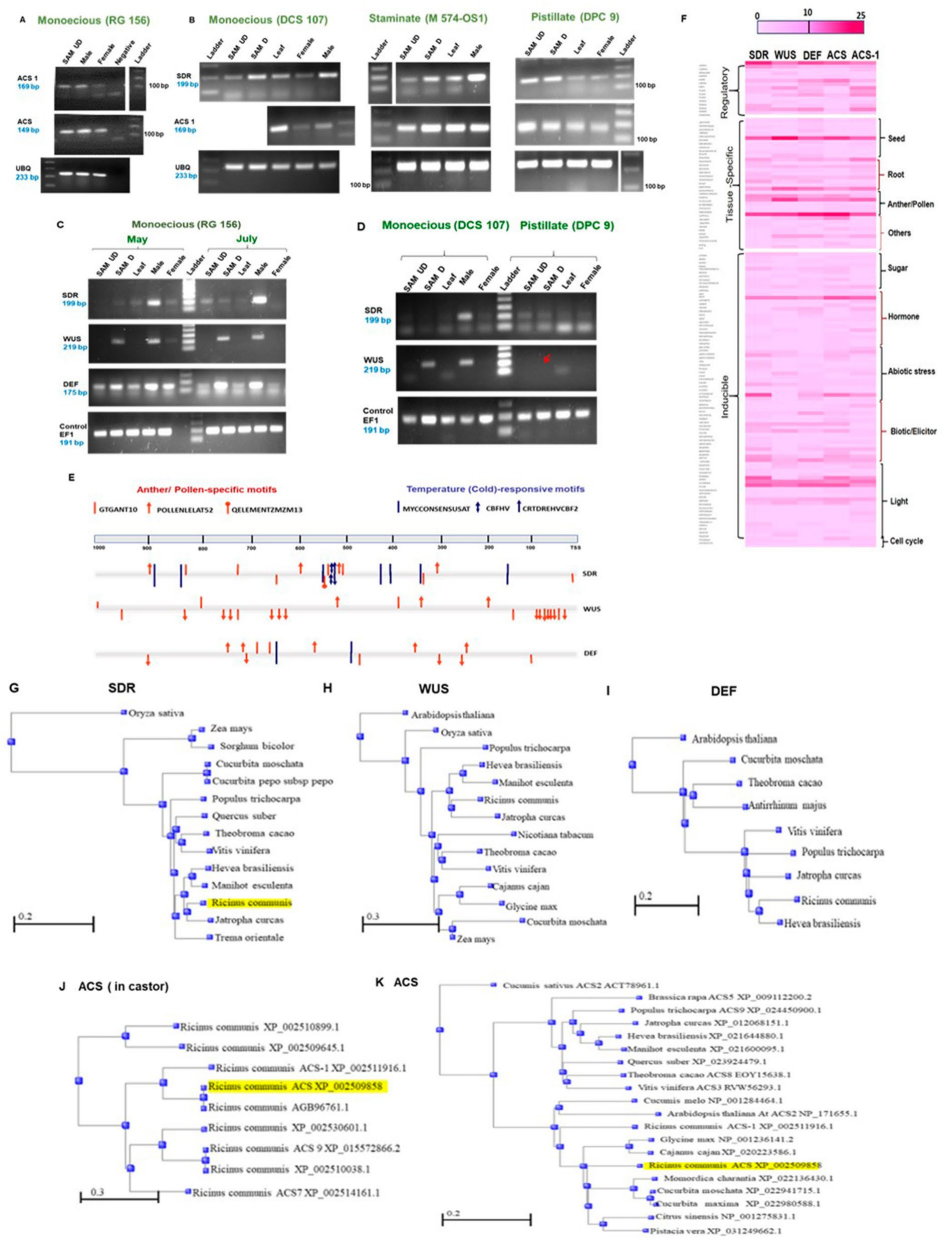
Gene expression analysis and in silico analysis of CREs of putative genes involved in sex expression of castor. (**A**) Expression analysis of ACC synthases *ACS* and *ACS-1* in monoecious RG 156. (**B**) Expression of *Short chain dehydrogenase reductase 2a* (SDR) and *ACS* in various castor genotypes. (**C**) Expression of *SDR*, *WUS* and *DEF* in monoecious RG 156 at high temperatures (38–41 °C) during May and at lower temperatures of 28–33 °C during July 2017. (D) Expression of *SDR* and *WUS* in monoecious DCS 107 and pistillate DPC 9 in 3 months-old crop during August-September, 2017. (**A**) and (**B**) show one-step RT-PCR experiments carried out during 2015-17 and (**C**) and (**D**) show two-step RT-PCR experiments conducted during 2018. RNA from vegetative or undifferentiated shoot apical meristem (SAM UD), differentiated SAM or inflorescence bud at stage II (SAM D), young primordial leaves from inflorescence bud (leaf), male flower buds (male) and female flower buds (female) were used for semi-quantitative RT-PCR. N is negative water control and L is 100 bp DNA ladder. DNA ladders for *ACS1* of RG 156 (in **A**), M 574-OS1 and *UBQ* of DPC 9 (in **B**) are shown separately. Amplicon length is indicated in blue below the gene name. *Ubiquitin* (*UBQ*) or *Elongation Factor-1 Delta* (*EF-1*) are internal control genes. Details of complete gels and plot profiles generated using ImageJ are given in Supplementary Data set 1: Supplementary Fig. S8). (**E**) Diagrammatic representation of anther orpollen-specific and temperature (cold)-responsive CREs in the 1 kb promoter region of *SDR*, *WUS* and *DEF* genes. Scale in bp is indicated in top bar. *TSS* is putative transcription start site. (**F**) Graphical representation of *cis*-regulatory elements (CREs) in the positive and negative strands of 1 kb promoter region of 5 differentially expressed genes as heat map. Colour code indicated at top from 0–25 represents number of motifs. CREs and their group or classes are indicated at left and functions of CREs are indicated at right. Phylogenetic trees of (**G**) SDR (**H**) WUS and (**I**) DEF of castor showing divergence from other genomes. Phylogenetic trees of (**J**) ACSs of castor showing divergence within castor and (**K**) ACS of castor showing divergence from other genomes. Scale is shown below.

Expression of 5 out of 17 candidate genes, were consistent and re-verified by semi-quantitative RT-PCR. Expression of 1-aminocyclopropane-1-carboxylate synthase (ACC synthase) genes, *ACS* and *ACS-1* were upregulated by 1.1-1.2 times, in male buds of monoecious genotype RG 156 (collected by May, 2015). Expression level of *ACS* was more than that of *ACS-1* in all the tissues (Fig. 8A). Expression of *SDR*) and *ACS-1* were compared in monoecious DCS 107, staminate M 574-OS1 and pistillate DPC 9 in samples collected by February–March, 2017. *Short-chain dehydrogenase reductase 2a *(*SDR*), a homologue of *Zea mays TASSELSEED2* (*TS2*), was expressed in all tissues such as vegetative and reproductive (differentiated) SAM, primordial leaves, male and female buds etc, but was highly expressed in male buds of monoecious genotypes DCS 107 (Fig. 8B) and RG 156 (Supplementary Fig. S7A). *SDR* expression was higher in male buds (by 1.4 times) than female buds of monoecious genotype DCS 107 and much higher in male buds of staminate genotype M 574-OS1 (by nearly 1.8 times) than that of monoecious genotypes, while feeble expression was noticed in female buds of pistillate DPC 9. Expression pattern of *ACS-1* was also similar to *SDR* in DCS 107, expression being higher in male than female buds (Fig. 8B).Samples were collected during 2nd week of May, when temperatures were as high as 41 °C and 2nd week of July (after summer when temperature drops to 32 °C) 2017 from monoecious RG 156 and expression of 6 control genes were verified at two temperature conditions by semi-quantitative RT-PCR. *Elongation Factor-1 Delta* (*EF1*) gene was found to be uniformly expressing, without much fluctuations, in all tissues at higher and lower temperatures and therefore chosen as the control gene for temperature studies (Supplementary Fig. S7B). Expression of various candidate genes were verified in the same samples. Expression of *SDR* was higher in male buds of RG 156, (by nearly 1.2 times) than female buds, while *WUSCHEL* (*WUS*) was expressed only in differentiated shoot apical meristem (SAM) that has undergone floral differentiation and in male buds, but was totally absent in vegetative tissues such as undifferentiated SAM and leaves (Fig. 8C). *DEFICIENS* (*DEF*), also showed higher expression in differentiated SAM and male buds, like *WUS*, but unlike *WUS*, expression of *DEF* was not totally absent, but lower in other tissues. Expression of *SDR* in male buds and expression of *DEF* in differentiated SAM and male buds were also higher in July, when the temperatures dropped down, than during May. However, expression of *WUS* did not vary significantly with temperature fluctuations, though feeble expression noticed in female buds  at high temperature conditions, was absent when temperatures lowered in July (Fig. 8C). The experiments were repeated and results re-confirmed (Supplementary Fig. S7C). Expression of *SDR* and *WUS* were verified in various tissues of monoecious DCS 107 and pistillate DPC 9 collected by August–September 2017 (at normal temperature of 32 °C) from 3-months old crop. In monoecious DCS 107, expression pattern of *SDR* and *WUS *were similar to that of RG 156, while in DPC 9, expression of *SDR* was feeble in all tissues, and almost absent from female buds and *WUS* expression was totally absent from all tissues including differentiated SAM, (Fig. 8D). The complete gels and plot profiles generated by ImageJ are given in Supplementary Dataset 1: Supplementary Fig. S8).

In silico analysis of the 1 kb promoter sequences upstream of the TSS of these 5 differentially expressed genes using PLACE detected a total of 105, 117, 133 putative CREs in positive, negative and both positive and negative strands respectively. Ten CREs such as mesophyll-specific CACTFTPPCA1 (YACT, S000449), endosperm-specific DOFCOREZM (AAAG, S000265), regulatory CAATBOX1 (CAAT, S000028), light-responsive GT1CONSENSUS (GRWAAW, S000198), pollen-specific POLLEN1LELAT52 (AGAAA, S000245), cytokinin-regulated ARRIAT (NGATT, S000454), light-responsive GATABOX (GATA, S000039), root-specific ROOTMOTIFTAPOX1 (ATATT, S000098), cold-responsive MYCCONSENSUSAT (CANNTG, S000407) and light-responsive EBOXBNNAPA (CANNTG, S000144) were predominant in the promoter regions of these genes, in the order of their predominance. Motifs responsive to plant hormones such as auxin, cytokinin, ABA, GA, ethylene and JA were present in these genes (Supplementary Dataset 2: Supplementary Table S6). The CREs in the 1 kb region for anther or pollen specific expression and temperature (cold)-responsiveness were found to associate in the *SDR* and *DEF* genes, but not for *WUS* (Fig. 8E). Similar association of CREs were observed for *ACS* genes which were upregulated in male buds of RG 156 collected during May 2015 (Supplementary Dataset 2: Supplementary Table S6).Heat map of different CREs in both strands arranged in functional clades in 5 differentially expressed genes shows their frequency (Fig. 8F) Motif for transcription factor LEAFY(LFY), LEAFYATAG was present in promoter sequences of *SDR* (94−) and *DEF* (386 +) and that for transcription factor WUS (WUSATAg was present in promoters of *DEF* (29−) and *ACS* (794 +), as well as in the intron 4 (1582 +) of *DEF* and intron 3 (1272−, 1697 +) as well as 5ʹ UTR (84−) of *ACS* genes. *SDR*2a and *WUS* were single copy genes, while *DEF* had two copies in castor viz., LOC8273018 and LOC8275809. *DEF* (LOC8273018) gene sequence  was 79.2% similar to *GLOBOSA* or *GLO* (LOC8270978), 78.15% similar to *AG* (LOC8267200) and 76.36% to AGAMOUS-like 104 or AGL104 (LOC8288528) of castor. DEF protein has 67–73.6% and 60% similarity to AGL104 and AGL65 proteins of castor respectively. Phylogenetic tree revealed that SDR and WUS proteins of castor were more related to those of *Jatropha curcas* than those of *Hevea brasiliensis* (Rubber) and *Manihot esculenta* (Tapioca), all the three species belonging to Family *Euphorbiacea*, while DEF of castor was more related to the ortholog in *Hevea brasiliensis* than *Jatropha curcas* (Fig. 8G–I). Divergence of eight ACSs (1-aminocyclopropane 1-carboxylate synthases) of castor genome are shown (Fig. 8J) ACS and ACS-1 were closely related. But ACS of castor was closely related to that of *Cucurbita sp* and less related to other members of *Euphorbiacea* (*Jatropha*, *Manihot* and *Hevea*) and to ACS2 of *Cucumis sativus* and *Arabidopsis thaliana* (Fig. 8K).

## Discussion

Genetic instability of females and unknown mechanisms of sex expression is a constraint in hybrid breeding programmes of castor^29^. To circumvent these constraints, inflorescence development and developmental mechanism of unisexual flowers of castor must be understood.

The inflorescence development in castor has eight morphological stages from floral initiation to capsule setting and takes 15–20 days till anthesis. In *Arabidopsis thaliana* 12 organogenic stages were identified, while floral meristem initiation to anthesis was divided into 12 and 16 stages in cucumber (*Cucumis sativus* L.) and *Fragaria* × *ananassa*, respectively^38–40^. Castor is protandrous (male flowers are formed first in monoecious raceme), but female flowers open first indicating the preference for cross pollination in castor, thereby contributing to wide genomic variations.

The inflorescence architecture controls number and size of seeds thereby affecting success of both seed and pollen parents^41–43^. In castor, the inflorescence architecture varied with and within genotypes in response to high temperatures. Architectural variation along the inflorescence may be due to decline in vascular tissue along the inflorescence length^44^ or due to hormonal gradient in the spike, determining the specific position of male, female or bisexual flowers in inflorescence. Auxin (IAA) has feminisation effect while ethylene, gibberellic acid (GA_4_) abscisic acid (ABA) and jasmonic acid (JA) have masculinization effect in castor^26,28,29,45^. The variation within genotype is phenotypic plasticity in response to different levels of resources or developmental constraints^46^. The flower number and size decrease in distal inflorescences, in response to decline in resource availability over time. The main axis becomes longer and produces more lateral flowers in racemose inflorescence^47^.

In the present study,  8 and 12 developmental stages were identified in male and female flowers of castor respectively. Similarly, in unisexual flowers of Cedreleae and in *Jatropha*, 12 developmental stages were identified^48,49^. The male and female unisexual castor flowers were morphologically similar upto stage 4, unlike flowers of *Jatropha* which were similar till stage 6^49^ or till stage 7 in *Vernicia fordii*^50^. Unisexual flowers of castor had an intermediate bisexual stage. Though bisexuality precedes unisexuality in castor flowers, the distinctness of bisexuality varied with genotypes, the pistil primordia being distinct in male flowers of M 574-OS1, but not in those of monoecious RG 156, while stamen primordia were distinct in female flowers of ISF line DPC 21, but not in those of pistillate line DPC 9. Earlier, rudimentary female organs were found to be absent in male flowers, but pistillate flowers were reported to have rudimentary stamens^51^. Female flowers of castor were believed to pass through bisexual stage^29^. We report here that both male and female flowers of castor have an initial bisexual state, the conspicuousness of which varied with genotypes and rudimentary female as well as male organs were observed in male and female flowers respectively. Rudimentary stamens occurred in female flowers, and rudimentary carpel was found in male flowers, but occurrence of bisexual flowers was occasional in castor.

In castor, sex is determined by selective growth arrest or abortion of either male or female reproductive organs, possibly through programmed cell death of opposite sex organs in bisexual primordia, similar to type 1 unisexual flowers^29,52^. Cells or primordia of both male and female organs arising initially, are selectively eliminated in female and male flowers respectively. Unisexuality but is the basic attribute in type 2 flowers^52^. Type 1 flowers are morphologically similar, while type 2 unisexual flowers, are morphologically distinct, where gynoecium and androecium are entirely absent from male and female flowers respectively^52^. Unisexual flowers of castor are morphologically distinct like that of type 2 flowers, with round male and elongated female flowers. Thus, unisexual castor flowers belong to an intermediate category between type 1 and type 2 flowers, having features of both. Castor flowers may be predominantly of type 1 category of flowers, since morphological distinctness is not precise in identifying sex of a flower, due to occurrence of slightly pointed male flowers and slightly round female flowers during summer.

In castor, sex determination takes place after an initial bisexual stage of each floral primordium, similar to maize and cucumber^2,53^. But whether programmed cell death (PCD) of preformed sex organs determines sex in castor^54^ is not known, though PCD-related cysteine protease gene was expressed at peak of anther abortion in pistillate line^29,55^. DNA methylation also suppresses male flower-specific gene expression in pistillate inflorescences^29^.

In castor, growth arrest of pistil (in male flower) occurs earlier than that of stamens (in female flower), the stage of inhibition being dependent on genotypes. Pistil abortion occurred earlier than that of stamen in cucumber and *Diospyros lotus*^39,56^, but later than stamen in *Asparagus officinalis*^57^. In castor, stamen inhibition is clear in pistillate flowers and can be traced as a layer of cells or small bulges at base of ovary, unlike in *Carica papaya* where pistillate flowers do not show any traces of stamens^58^. Sex differentiation occurs at different stages after floral meristem development, such as flower organ primordia initiation (by developmental arrest of both stamen and pistil), organ differentiation, pre-meiotic and post-meiotic stages (by selective degeneration of opposite sex organ in unisexual flowers of only one sex type, but not the other)^36^. Sex differentiation in castor flowers occurs by growth arrest of opposite sex organs at stage 6 during organogenesis before meiosis.

Stamens can develop at a later stage after initial inhibition as seen in reversion to bisexuality in female flowers, but not pistil. After inhibition, pistil primordia do not develop into fully functional ovary, but can only develop simultaneously with stamens. Larger anthers in rudimentary stamens of bisexual flowers suggest incomplete divisions, where all developmental steps of male programming are not present. Similarly, rudimentary pistil with under-developed ovary and tubular style-like elongated structure results when female developmental programme is incomplete, altered and inhibited. Thus, growth arrest of the inappropriate organ does not uniformly affect the organ but occurs only in portions of the organs and all the arrested portions are spore-bearing parts^39^.

In monoecious plants, male as well as female programs, genes and signals co-exist in all cells and the sexual fate of cells is determined by sex-determining genes, their interaction within the cells and external factors, but there are no sex-determining loci^59,60^. Thus, all individuals are capable of developing flowers of either sex, where interaction and regulation of the sex-determining genes in response to environment results in sex-phenotype variations^60,61^. This explains for the sex lability and high sexual polymorphism observed in castor. Of the various factors affecting sex expression, high day temperature (T_max_) was found to be major cause of sex alterations and reversions. Extreme temperature events (5 °C above the normal temperatures) of short-term durations may have adverse impact on plant productivity^62,63^. Low temperature favoured female flower differentiation, while high temperatures changed in inflorescence morphology and increased the proportion of staminate to pistillate flowers (4:1) in plants^64,65^. Plant growth and development are more associated to the thermal than chronological time and degree day’s approach is widely used for thermal time quantification. In castor, sex phenotype alterations and sex reversions observed at extremely high day temperatures (above 33–34 °C) and degree days (above 19–20) during floral differentiation stage, was unidirectional towards maleness at high temperature, but femaleness was restored at lower or normal temperatures, indicating the role of temperature in determining sexuality and sex reversibility of castor flowers. High temperature causes maleness^22^. Reproduction is resource-intensive, females use more resources for reproduction than males^42^ and female development pathway acts downstream to male developmental pathway. Therefore, female flower developmental program is entirely shut down and male flower development program requiring minimal resources is favoured, under high temperature stress conditions. Other factors such as order of branching, genotype (genetic factors) and physiological factors also influence sex expression. Ethylene, a stress hormone is produced under high temperature conditions. Auxin level at the differentiating apex which determines the sex balance of the flowers is in turn influenced by temperature, nutrition and photoperiodism^24^. Male flowers could be induced even at low temperatures of 30 ºC by removing female flowers^66^. The alteration in sexuality in castor is due to the effect of temperature rather than the higher branch order or ageing that occurs during the advancement of growing season, since femaleness is restored even at higher orders of branching, when temperature lowers after summer. The floral developmental pathway in unisexual flowers not only varies with temperature, but also with the genotype, since female flowers of DPC 21 had more distinct stamen primordia even at lower temperature conditions and later stages of development, when compared to DPC 9. Bisexuality is not a norm since bisexual flowers with equally developed male and female organs do not occur in all, but only in few genotypes, indicating that out-crossing results in genetic instability that alters developmental programming in inflorescence. Control of sex expression is thus frequently the control of floral organ development, stopping or starting an existing, functional developmental pathway^67^.

In monoecious plants, the successive induction of first male (protandrous) and then the female program, results from inverse gradients of male and female signals^59^. It is ‘male first’ programming in castor at inflorescence and individual flower level. Female organ development is more complex, requiring activation of more genes or regulatory networks for ovary, stigma and ovule development and for silencing genes of male organ development. More genes were expressed close to raceme formation in pistillate line when compared to monoecious line^29^. Genes involved in development of female organs act downstream to male organs but not simultaneously. A few critical or unique female development genes may act after stamen inhibition in female flowers or after activation of male developmental programme in bisexual flowers. Some genes in male and female flower development may have overlapping functions or crosstalk. Gene expression analysis shows that *SDR*, a *short chain dehydrogenase reductase 2a* was expressed in all tissue samples. but higher expression of *SDR* in male than female buds and in male buds of staminate than monoecious genotypes, indicates its significant role in pistil abortion in male flowers and maintenance of maleness, in otherwise bisexual floral primordia. *SDR* expression was lower at high temperatures of summer (May) and higher when temperature drops down in July, in male flowers. This may be because of general deregulation or lower expression levels of genes at extremely high temperature. Lower temperatures favour femaleness or pistil development, and hence *SDR* expression increases at low temperatures in male flowers, for abortion of the pistil primordium, thus maintaining maleness in male flowers and spikes of staminate genotypes. Even when bisexuality was observed in staminate genotypes at lower temperatures during June-July, the expression of *SDR* is still low in female flowers and increases in male flowers of monoecious genotype, indicting its significance in male flower development. Short-chain dehydrogenases/reductases (SDRs) constitute one of the largest and oldest protein superfamilies found in all domains of life, having 49 different families and  are classified into 5 different types (classical, extended, intermediate, divergent and complex) , the diversified ones are involved in secondary metabolism and developmental processes, while the less diversified ones are involved in primary metabolism^68^. *SDR2a* in castor is a homologue of *TASSELSEED2* (*TS2*) of maize, a short-chain alcohol dehydrogenase or hydroxysteroid dehydrogenase, which determines sexual fate of floral meristems in maize through sub-epidermal expression in the gynoecium primordium of male flowers, resulting in pistil abortion through programmed cell death^54^. Though all pistil primordia in maize express *TS2* RNA, *TS2*-induced cell death in functional pistil primordia of primary ear florets is blocked by the *silkless1* gene^69^. *TS2* is expressed throughout the plant in maize, rice and sorghum and the expression of its homologues in different species vary such as in tapetal cells of male flowers in *Arabidopsis thaliana* and *Silene latifolia* and in all sampled tissues of grasses, suggesting that *TS2* may not be involved in sex determination and may have a general developmental role^67,70^. *SDR2a* in castor, similar to *TS2* in grasses is single copy, but there are many putative *SDR*s and secoisolariciresinol dehydrogenases in castor which belong to the same class as *SDR2a*. Although the stamen and pistil abortion for female and male flower development are under two different genetic pathways, considering the wide morphological diversity of sexuality and flowers in castor, *SDR* is unlikely the single master switch affecting transition of sexuality, suggesting the role of multiple genes in sex determination. *SDR2a* in castor may be one major gene player involved in sex determination in castor and with multiple roles, as indicated by the presence of CREs for light-responsiveness and inducibility to abiotic and biotic stresses as well as elicitors in its promoter region.

*WUSCHEL* (*WUS*) is a homeobox gene encoding homeodomain transcription factor required for the maintenance of stem cell homeostasis in the SAM and floral meristem (FM), expresses in a few cells in the L3 layer or stem cell–organizing center (OC) and is antagonized by *CLAVATA* (*CLV*) signaling^71,72^*. WUS* flowers display many fewer stamens (usually one or two) and no carpels, consistent with precocious FM termination^73^*. WUS* orthologue in castor is a single copy gene without any paralogues, expressed only in differentiated SAM and male buds, but absent from vegetative SAM, leaf and female buds of monoecious genotype and absent even from the differentiated SAM in pistillate genotype DPC 9, suggestive of its role in male floral development pathway. Expression of *WUS* in castor was similar to *ROSULATA* (*ROA*), a *WUS* homologue in *Antirrhinum*, where higher expression was noticed in inflorescence and stamens while expression was absent from stem and leaf and low in vegetative apex, petal and carpel^71^. WUS was expressed in SAM in *Arabidopsis*, in young leaf primordia in rice, and in reproductive meristems in maize^74^. Also, *WUS* expression did not vary with temperature fluctuations, which is also supported by absence of temperature-responsive CREs associated with anther or pollen-specific motifs in the 1 kb promoter region. WUS is a transcription factor which can act as a repressor as well as an activator. WUS represses certain genes in the SAM but activates them in the floral meristem (FM). *WUS* and *AGAMOUS* (*AG,* a class C homeotic gene in *Arabidopsis*) feedback loop starts with the activation of *AG* transcription at stage 3, and ends with the repression of *WUS* (by *AG*) in the centre of the FM, at stage 6 after carpel initiation, thus maintaining the WUS expression within the FM organizing centre^73^. Absence of *WUS* expression in female buds of castor indicates that floral meristem is not active after carpel primordia initiation which is also suggestive of parietal placentation in castor where placenta and ovules develop after the FM has terminated^73^. WUS binds to WUSATAg target sequence in the regulatory intron of *AG*, which causes repression of WUS in SAM and activation of *AG* in the floral meristem. Presence of WUSATAg sequence near to TSS (at 29-) and intron 4 (1582 +) of *DEF* and close sequence similarity of DEF with AG gene (78.15%), indicates overlap of functions *DEF* and *AG* genes of castor and that *WUS* may regulate *DEF* as well. *DEF* and *AG* of castor are closely related. Preliminary analysis of expression pattern of *AG* in castor showed upregulation of *AG* in differentiated SAM or inflorescence bud, female buds of monoecious and pistillate genotypes and male buds of staminate genotype (data not shown). *DEFICIENS* (*DEF*), a class B homeotic gene is necessary for development of petals in whorl 2 and stamens in whorl 3, along with *APETALA1* (*AP1*) and *AG* respectively. Upregulation of *DEF* in differentiated or reproductive SAM and male flowers of castor is similar to that of expression pattern of *WUS* in castor, which however shows expression only in these. DEF A protein controls floral organogenesis in *Antirrhinum majus* and expression of *DEF* orthologues also varied in different species such as *pMADS1* of petunia, a petal organ identity gene, expressed strongly in petals, but moderately in stamens, in root nodules in *Medicago*, in developing fruit of tomato, and in four floral whorls as well as in the leaves of orchid^75,76^. The class B gene (*AP3*) in a monoecious, diclinous species *Vernicia fordii*, was significantly increased in male flowers^50^. Comparative approaches in the MADS box gene family have provided some evidence that basic principles of the ABC model of flower development such as B-function genes are conserved in angiosperms^75^. *GLOBOSA* (*GLO*) is another class B gene and its homologue in castor (Gene ID: 255558565; XM_002520262.1) was found to be differentially expressed during raceme formation^29^. *DEF* and *GLOBOSA* (*GLO*) are hypothesised to have originated from a duplication event and duplication of *DEF/GLO* genes is an ongoing process^76^.

ACC (1-amino cyclopropane-1- carboxylate) synthases are responsible for production of plant hormone ethylene. Nine ACC synthases were identified in castor. Expression pattern of two paralogues *ACS* and *ACS-1* were similar, with higher expression in male than female buds, indicating its role in promoting maleness. Ethylene and ethylene-like substances promote maleness and can transform female flowers into male ones in monoecious plants^26^. In cucumber, ethylene promotes female flower by inhibition of stamen development, through organ-specific DNA damage in the primordial anther of female cucumber flowers^77,78^. Downregulation of ethylene receptor in stamens of female flowers, carpel-dependent expression patterns of pre-miRs and M gene (*CsACS2*) in cucumber its ortholog in melon A gene (*CmACS7*) etc. were involved unisexual flower development^78–80^. In castor, whether organ-specific DNA damage in gynoecium of male flowers is triggered by ethylene and whether the ethylene receptors are differentially expressed or downregulated in pistil primordium of male buds is not clear. Although gene expression pattern and levels may not exactly reflect the functions of the genes, the variable expression pattern in the paralogues and orthologues of these genes in different species suggests the multiple functions of the ancestral genes and conservation of imperative functions during evolution of these genes. In situ hybridisation on SAM and floral meristem can further reveal the detailed expression of these genes. We report here 5 male-specific or male abundant genes in castor, viz., *SDR2a, WUS, DEF*, *ACS* and *ACS-1*, which may be involved in sex determination. A MYB-like gene, Male Specific Expression 1 (MSE1), specifically expressed in males in early anther development and tight linked with the Y chromosome, acts in sex determination in dioecious *Asparagus officinalis*^81^.

*SDR* and *DEF* in castor male flowers are regulated by temperature as evidenced by their expression pattern and presence and close-association of temperature-responsive elements with the anthe or pollen-specific motifs in the 1 kb promoter region.*ACS* genes also show similar association of CREs, indicating their temperature responsiveness. However, *WUS* was devoid of such temperature regulation of spatial expression. A predicted CRE in the 1 kb upstream region is potentially diagnostic of the regulatory function of that CRE^82^ and hence we had chosen 1 kb region upstream of TSS for our analysis. Ten CREs viz., CACTFTPPCA1, DOFCOREZM, CAATBOX1, GT1CONSENSUS, POLLEN1LELAT52, ARRIAT, GATABOX, ROOTMOTIFTAPOX1, MYCCONSENSUSAT and EBOXBNNAPA were found to be predominant in the differentially expressed genes which were male-specific or male-abundant showing higher expression in male buds and staminate genotypes. Seven of these elements DOFCOREZM, CAATBOX1 GT1CONSENSUS, POLLEN1LELAT52, GATABOX, ROOTMOTIFTAPOX1, EBOXBNNAPA and other elements WRKY71OS, GTGANTG10 (pollen-specific), ACGTATERD1 and YACT were reported as 11 representative common elements in the 140 male gamete- and tapetum-expressed genes in rice with a frequency from 51.2 to 86.3%^83^. DOFCOREZM, CACTFTPPCA1 and CAATBOX1 were also the most abundant CREs in 414 Putative Promoter Regions (PPRs) of MLO (powdery Mildew Locus O) genes in plants^84^. Mere presence or predominance of certain *cis*-regulatory elements or motifs (CREs) in the promoter regions of genes do not necessarily imply their biological role. Some motifs such as ACGT may be frequently present in most plant gene promoters. The predicted or putative CREs could be short motifs that occur randomly throughout the genome without regulatory function and their biological significance can be determined through combinatorial or independent action of CREs by correlating their presence or absence to the expression profile of the genes^82^. The *cis*-regulatory codes specifying pCRE presence and absence, combinatorial relationships, location, and copy number were used to predict stress-responsive expression^85^. The *cis*-regulatory fingerprint helps in understanding the regulation of genes under various conditions. Presence of hormone responsive CREs in putative promoter regions of these genes are indicative of their regulation by plant hormones. 15 hormone-related genes involved in abortion of stamen during pre-meiosis of female flowers were identified in *Litsea cubeba*^86^.

Abnormalities in meiotic segregation of chromosomes or trisomy^12^ may also result in sex phenotype alterations (male racemes with rarely occurring single terminal or few hermaphrodite flowers) in outcrossed pistillate lines. However, preliminary analysis of the mitotic chromosome number in different genotypes with distinct sex expression pattern like monoecious, ISF and pistillate, did not show any significant variation in chromosome number (2n = 20) (Parvathy et al. unpublished). Genomic instability due to outcrossing could also be mediated by repetitive DNA or transposons (> 50% of genome) or epigenetic changes associated with these repetitive sequences, which are abundant in castor^87^.

## Conclusions

Flowering and sexuality are complex processes and end results of interactions and combinations of multitude of factors such as genetic, molecular, epigenetic, cytogenetic and environmental factors. The study was undertaken to understand evolution of unisexuality in castor flowers. Floral development pathways, sex determination stage (stage 4) and an intermediate bisexual stage (conspicuousness of which varied with genotype and temperature) were delineated and first time reported in both male and female flowers in castor. Sex expression of a raceme in castor is labile, determined by a combination of internal (hormones) as well as environmental factors persisting during the cropping season and coinciding with spike initiation or development, especially at stage 4 of flower development. Elevated day temperatures during stage 4 can favour male programming and alter sex expression in almost all the genotypes, hence for desired sex phenotype, chemicals, hormones or other biotechnological tools can be employed when maximum number of flowers are at stage 4. The complex phenomenon of sex reversion in pistillate lines of castor is due to alteration in the developmental pathways of male and female flowers, a major mechanism governing sex expression and reversion. The alteration in sexuality is because of temperature rather than the branch order or ageing. Reversion or alteration of sexuality such as male to bisexual to female in flowers is also first time reported to change with temperature. Duration of thermal stress existing during critical stages for 2–3 weeks during inflorescence or floral meristem initiation and sudden variations (rise or drop) in temperatures alter sex expression, rather than the absolute value of T_max_. In addition to temperature, genetic factors also contribute to sex variations, since completely male inflorescences were observed even during normal temperatures of early growing season in the primaries of DPC9-OS2. Two genes, *SDR2a* and *WUS* (orthologs of *TS2* of maize and *WUS* of *A. thaliana* respectively) were male-abundant and male-specific, the other three male-abundant genes being *DEF, ACS* and *ACS-1*. Knock out or silencing of these male organ-predominant or male-specific genes in bisexual stage of flowers by use of suitable promoters that drive expression during the inflorescence or floral bud initiation, will result in fully female flowers and there will be no revertants. Transgenics with knock out of these male-abundant or male-specific genes can be developed for desirable stable sex phenotype such as completely pistillate spikes or plants without reversion or ISF, even at summer. Also, the *cis*-regulatory regions or promoters of the male-specific genes can be cloned and used to drive expression of male-sterility or other genes in male flowers and used as additional tools for developing transgenics with desirable traits. The differentially expressed genes identified can be used as functional markers in marker-assisted selection or the gene sequences used in gene chips to identify male specificity. Understanding the morphogenetic and molecular regulation of floral developmental pathways will thus enable in devising effective chemical or biotechnological tools as well as strategies to regulate sex expression in castor.

## Methods

### Plant material

DCS 107 (monoecious variety with lower 3–4 whorls or 30–50% male flowers), RG 156 (monoecious germplasm accession with 70–80% male flowers), DPC 9 (completely pistillate line), DPC 21 (pistillate line with interspersed staminate flowers or ISF), DPC 17-S3 (selection from pistillate line DPC 17; monoecious with apical male flowers or apical ISF), DPC 16 (pistillate line with bisexual flower), M 574-OS1 (selection from outcrossed population of M 574 having monoecious inflorescences with tip bisexual flower in lower branch orders and completely male inflorescences with tip male flower in higher orders above tertiary or quarternary) and DPC 9-OS2 (selection from out-crossed progeny of pistillate DPC 9, having staminate inflorescence with few female and bisexual flowers, similar to that of male M 574- OS1), were used for the study. The various castor genotypes, including pistillate and male parental lines, varieties and germplasm accessions used in the study are enlisted (Supplementary Table S7).

The plants were grown in open conditions in pots of 28 cm diameter containing red soil, black soil and farmyard manure in 2:1:1 proportion, for histology studies. The plants were also raised in the field at ICAR-IIOR (Rajendranagar, Hyderabad, Telangana, India) at row spacing of 90 × 90 cm with ten plants per row for studies on histology, scanning electron microscopy (SEM), inflorescence growth, stage transitions and inflorescence architecture. The castor genotypes were observed for sex expression in different orders at ICAR-IIOR (Rajendranagar, Hyderabad, Telangana, India) at least for two seasons (2012–2014) before collecting samples.

### Inflorescence growth and architecture

The inflorescence buds at different stages after differentiation before and till complete emergence from bracts, located at the apex of different branch orders (secondary to quaternary) were tagged in field-grown plants of DCS 107, RG 156, DPC 9, DPC 21 and DPC 17-S3. The number of buds tagged for studies on inflorescence growth and stage transition are shown in Supplementary Table S8. Inflorescence growth and morphological stage transitions were monitored at regular time intervals of 3–4 days for 2–4 weeks. Inflorescence length (in centimetres, cm) was measured using a 30 cm metallic scale, the mean of observations at each stage calculated, and the growth tendency of inflorescence depicted in timeline graph. Number of days taken for transitions to each stage was recorded and the days to anthesis noted for each genotype.

For understanding the floral architecture, the inflorescences at different growth stages after complete emergence, but before anthesis were harvested from different branch orders, (secondary to quaternary) from field-grown plants of DCS 107, RG 156, DPC 9, DPC 17-S3 and M 574-OS1. The length of the inflorescence (in cm) was recorded and different floral whorls were removed in the order from bottom to top of inflorescence and arranged such that, bottom-most was first whorl and top-most was last whorl. The total number of floral whorls in each spike and flower buds (male and female) in each whorl with their positions were recorded and represented diagrammatically. The whorl number (counted from bottom of spike) in which female flower buds first appear and whorl number where all flower buds are female were noted for monoecious lines and whorl with male flowers in DPC 17-S3 (monoecious apical ISF) and M 574-OS1 were noted. To know whether floral whorls are added during elongation of inflorescence, pearson’s correlation coefficient was calculated, for length of inflorescence and number of floral whorls, One-tailed t-test was performed (alpha = 0.05, 95% significance level) and compared with table value (t_critical_). Similarly, calculated r was compared with the table r value (at alpha = 0.05). A relationship existed between inflorescence length and number of floral whorls when calculated value > table or critical value and p < 0.05. Data analysis was carried out using MS Excel version 2010.

### Histology and light microscopy

DCS 107, RG 156 and DPC 9 were used for sectioning or histology. Samples consisting of growing tips or inflorescence buds (before emergence of inflorescence from bracts) of the primary branch at different growth stages of the plants, as determined by node numbers (from 5th to 20th node, 3–5 buds per node), were collected from pot-grown or field-grown plants at ICAR-IIOR (Rajendranagar, Hyderabad, Telangana, India) over a 1-year period (2013–14) in FAA (10 ml formaldehyde, 5 ml acetic acid, 50 ml alcohol solution and 35 ml distilled water in 100 ml solution) and fixed overnight at room temperature or stored in FAA at 4 °C till use. The samples were rinsed with distilled water to remove fixative, dehydrated in graded series of ethanol [10, 30, 50, 75, 90, 100, 100% (with a drop of toluidine blue for staining the specimen)], two xylene treatments each for 1 h, infiltrated twice in paraffin wax, each for 1 h and embedded in wax. Wax blocks were prepared using hand-made paper boats and serial sections of 5 µm thickness were taken using hand-operated microtome (Leica RM 2245, Lab India). The wax ribbons were gently placed on labelled, preheated slide smeared with Mayer's adhesive (1:1 v/v egg albumen: glycerol). The slides were dewaxed with xylene, placed in graded series of ethanol (100, 95, 70, 50 and 20%), stained with 0.7% toluidine blue, dehydrated in graded series of ethanol in the reverse order (20, 50, 70, 95, 100, and 100%), air dried and mounted with D.P.X. mountant (Fischer Scientific, Mumbai, India). At least 3 biological replicates were included per node and 4–5 biological replicates were used in case of DCS 107 and DPC 9. Minimum 10 slides with 2 rows of sections (20 technical replicates) were analysed per specimen. Microtome sections were observed and photographed using a compound light microscope (H600L, Nikon, Tokyo, Japan) at 40X magnification. The images were captured in a series of frames and superimposed to reconstruct the whole section of the specimen.

### Scanning electron microscopy

M 574-OS1 (completely male inflorescences with tip male or bisexual flower in higher orders above quaternary) and DPC 9 (completely pistillate) were used for studying male and female flower developmental stages respectively. In addition, RG 156 (monoecious), DPC 9-OS2 (staminate inflorescence with few female and bisexual flowers), DPC 21 (ISF) and DPC 16 (terminal bisexual flower) were used to probe bisexuality and variations in male and female flower development. Samples (growing tips or inflorescence buds at different growth stages covered with bracts) were collected from the primary or higher order branches of plants grown in field at ICAR-IIOR (Rajendranagar, Hyderabad, Telangana, India) over a two-year period (2014–2016) in 2.5% glutaraldehyde in 0.1 M phosphate buffer (pH 7.2) and fixed for 24 h or till use for a week at 4 °C. After removing outer whorl of bracts and leaves covering inflorescence, bracts covering the shoot apical meristem and individual flower bud primordia were carefully removed, the inflorescence bud trimmed and flower buds at different growth stages cut open with scalpel or needle as and when required, under a SM Z800 Nikon stereo zoom microscope. Nearly 500 buds were dissected in each genotype. The samples were post fixed in 2% aqueous osmium tetroxide for 4 h, dehydrated in a graded series of alcohol, dried to critical point drying with Electron microscopy science CPD unit and further dissected as required. The processed samples were mounted on metallic stubs with double-sided carbon conductive adhesive tape, sputter coated with gold using an automated sputter coater (JEOL-JFC 1600) for 3 min and scanned under Scanning Electron Microscope (JEOL-JSM 5600, Cambridge, UK) at 10 kV at various magnifications as per the standard procedures and images captured. Steps from post fixation with osmium tetroxide were done at Ruska Labs, College of Veterinary Science, Sri P.V. Narasimha Rao Telangana State University for Veterinary and Fisheries Sciences (SPVNRTSUVAFS), Rajendranagar, Hyderabad, Telangana, India. Alternatively, few samples collected during 2016 were fixed in methanol, trimmed, dehydrated in graded series of ethanol (70, 90, 100 and 100%) treated with hexamethyldisilazane (Sigma-Aldrich) twice for 20 min, decanted and the samples dried overnight in a desiccator at room temperature. The samples were mounted as mentioned above, sputter coated with gold using sputter coater (Q150R ES, Quorum Technologies Ltd, East Sussex, UK) for 3 min and scanned at 10–20 kV using scanning electron microscope (Philips XL30) at Central Instrumentation Facility, University of Hyderabad, Telangana, India and images captured at various magnifications.

### Weather data

Weather data on daily minimum and maximum temperatures, rainfall and humidity at Rajendranagar region (17.3203° N, 78.4018° E) of Hyderabad, Telangana, India, from January 2014–July 2017 were obtained from Agroclimatic Research centre, Agricultural Research Institute, Professor Jayashankar Telangana State Agricultural University (PJTSAU), Hyderabad, Telangana, India. Growing degree days (GDD) was calculated for the cropping seasons of 2014–15, 2015–16 and 2016–17 (date of sowing as starting date), using the average method^88^, GDD = [T_max_ + T_min_)/2] – T_base_, where T_max_ is daily maximum temperature, T_min_ is daily minimum temperature and T_base_ was base temperature with standard value 10° C. The date of sowing or planting were 24 July, 2014, 22 July, 2015 and 12 August, 2016 respectively for the cropping seasons 2014–15, 2015–16 and 2016–17 respectively and days after planting (DAP) calculated accordingly. Cumulative degree days (CDD) for the cropping season was calculated by adding the consecutive values for degree days, starting from date of sowing. All the weather parameters for the cropping season (GDD, CDD, T_max_, T_min_, T_avg_) were represented graphically using MS Excel 2010 and related with the variations in sex expression in the genotypes during the growing season in the field. Effect of temperature on castor inflorescence was studied  in new flushes of eight castor genotypes after irrigation during summer of 2015–16 and 2016–17 and thereafter, when temperatures dropped down during June-July 2017. Mean of the degree days, T_max_, T_min_ and T_avg_ were calculated for 2–3 weeks before sample collection for scanning electron microscopy and sex reversions or variations in sex phenotype correlated with these values.

### Semi-quantitative RT-PCR

Primers were designed manually for 5 candidate genes* in castor (based on information on their role in sex determination in other crops) and six control genes (Supplementary Table S9). PCR conditions were standardized using gradient PCR. RNA was isolated from different tissues or stages in various genotypes such as monoecious (DCS 107, RG 156), pistillate DPC 9 and staminate M574-OS1 using Trizol^®^ method during 2015–2018. Atleast three biological replicates per sample (SAM undifferentiated or differentiated, primordial leaf etc.). were pooled for RNA isolation. SAM differentiated was inflorescence bud at stage II. Equal number of male and female buds were taken. The quality and quantity of isolated RNA were verified using agarose gel and Nanodrop^®^ spectrometer respectively, DNase I (Genetix)- treated and equal quantity of RNA (after adjusting volume according to the gel intensity or nano drop readings) was directly used in one-step RT-PCR using PrimeScript™ One-step RT-PCR kit (Takara Bio) for verification of expression of control (*EF-1* and *UBQ*) and/or candidate genes as per manufacture’s protocol. Alternatively, first strand of cDNA was synthesised using Superscript™ III (Invitrogen) as per the standard protocol and used in two-step RT-PCR to verify the equal expression of control gene in all samples. The same quantity of cDNA used to normalise control gene expression, was used for verifying expression of candidate gene in all the samples with 0.4–0.6 µM forward and reverse primers, 0.4 mM dNTP, 1 U of Taq DNA polymerase (Merck), 2 µl cDNA (1: 20 dilution) using the programme, initial denaturation at 94 °C for 3 min, 33 cycles of 94 °C for 1 min 50–60 °C based on T_m_ of primersfor 1 min, 72 °C for 1 min and final extension at 72 °C for 10 min, in Eppendorf Mastercycler^®^ Nexus gradient PCR master cycler. The RT-PCR products were run on 1% agarose gels stained with ethidium bromide and the intensity of bands were quantified using ImageJ (https://imagej.nih.gov/ij/) [*Primers were synthesized for 17 candidate genes and gene expression verified in various genotypes, but based on consistency of results and reconfirmations 5 are reported].

### In silico analysis of differentially expressed genes

The 1 kb sequence upstream to the putative or annotated TSS and the gene sequences of the differentially expressed genes were extracted from NCBI (ncbi.nlm.nih.gov) and used for identifying *cis*-regulatory elements (CREs) using New PLACE (https://www.dna.affrc.go.jp/PLACE/?action=newplace)^89^. The CREs in the 1 kb putative promoter region of the genes were categorised into classes based on their function, their occurrence counted in the positive and negative strand and a heat map drawn using MS Excel version 2010. The gene sequences from NCBI-GenBank and protein sequences extracted from UniProtKB (https://www.uniprot.org) were used for nucleotide as well as protein blast, carried out using NCBI-BLAST (blast.ncbi.nlm.nih.gov) and phylogenetic trees were constructed using the BLASTp results by neighbour joining method.

## Supplementary Information

Supplementary Dataset 1.

Supplementary TableS6.

Supplementary Information.

## Data Availability

Relevant data generated or analysed during this study are included in this published article (and its Supplementary Information files). Data are however available from the first author, who is presently at ICAR-Indian Institute of Agricultural Biotechnology (ICAR-IIAB), Ranchi, India upon reasonable request and with permission of ICAR-IIOR, Hyderabad, India.
