# Author Correction: Probing the floral developmental stages, bisexuality and sex reversions in castor (*Ricinus communis* L.)

**DOI:** 10.1038/s41598-021-89718-y

**Published:** 2021-05-12

**Authors:** Sujatha Thankeswaran Parvathy, Amala Joseph Prabakaran, Thadakamalla Jayakrishna

**Affiliations:** 1 ICAR-Indian Institute of Oilseeds Research, Rajendranagar, Hyderabad, Telangana 500030 India; 2 Present Address: ICAR-Indian Institute of Agricultural Biotechnology, Ranchi, Jharkhand 834010 India; 3 Present Address: ICAR-Sugarcane Breeding Institute, Coimbatore, Tamil Nadu India

Correction to: *Scientific Reports* 10.1038/s41598-021-81781-9, published online 19 February 2021

This Article contains errors.

In the Results section under the subheading ‘Differentially expressed genes may determine sexuality in castor flowers’,

“Divergence of eight ACSs 1-aminocyclopropane1- carboxylate synthases) of castor genome are shown (Fig 8J)”

should read:

“Divergence of nine ACSs (1-aminocyclopropane1- carboxylate synthases) of castor genome are shown (Fig 8J)”

Additionally, this Article contains errors in the Acknowledgements section.

“We are grateful to the Indian Council of Agricultural Research (ICAR), New Delhi, India for providing fnancial support to the institute project DOR-103-11 (PIMS No: IXX09329) ’Elucidating the olecular mechanisms governing sex expression in castor (*Ricinus communis* L.)”

should read:

“We are grateful to the Indian Council of Agricultural Research (ICAR), New Delhi, India for providing financial support to the institute project DOR 103-11 (PIMS No: IXX09329) 'Elucidating the molecular mechanisms governing sex expression in castor (*Ricinus communis* L.)”.

