# Supplementary material for: Probing the floral developmental stages, bisexuality and sex reversions in castor (Ricinus communis L.)
Source: Sci Rep. 2021 Feb 19;11:4246. doi: 10.1038/s41598-021-81781-9 (PMC7895920; doi:10.1038/s41598-021-81781-9)
Supplement: Supplementary file 1 — Supplementary Dataset 1. [file 41598_2021_81781_MOESM1_ESM.pdf]

Probing the Floral Developmental Stages, Bisexuality and Sex Reversions in Castor (*Ricinus communis* L.)

Scientific Reports

Sujatha Thankeswaran Parvathy[1*], Amala Joseph Prabakaran[2] and Thadakamalla Jayakrishna[3]

ICAR-Indian Institute of Oilseeds research, Rajendranagar, Hyderabad, Telangana, 500 030 India.

*E-mail: sujatha.parvathy@icar.gov.in, hiisuj1@gmail.com

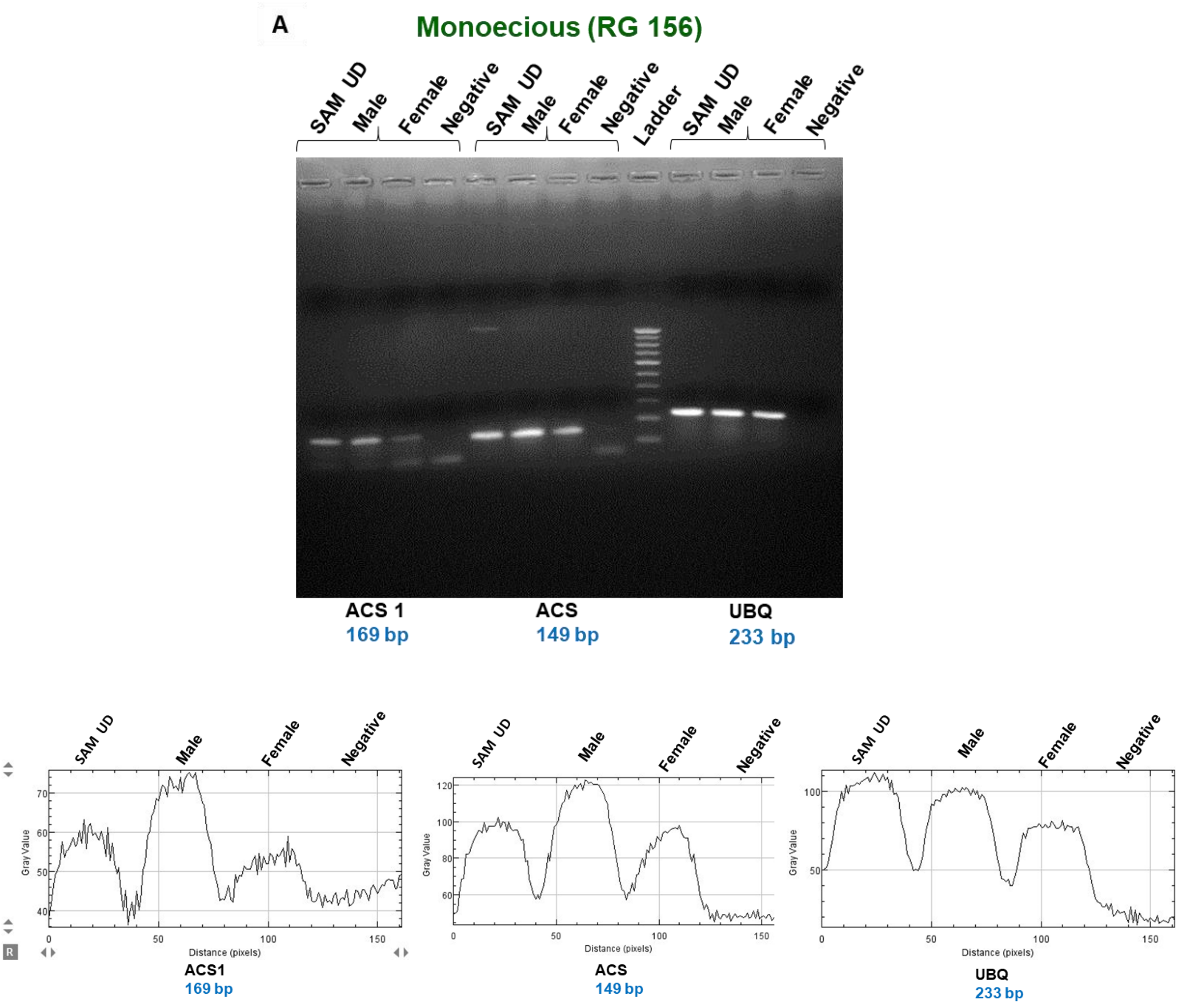

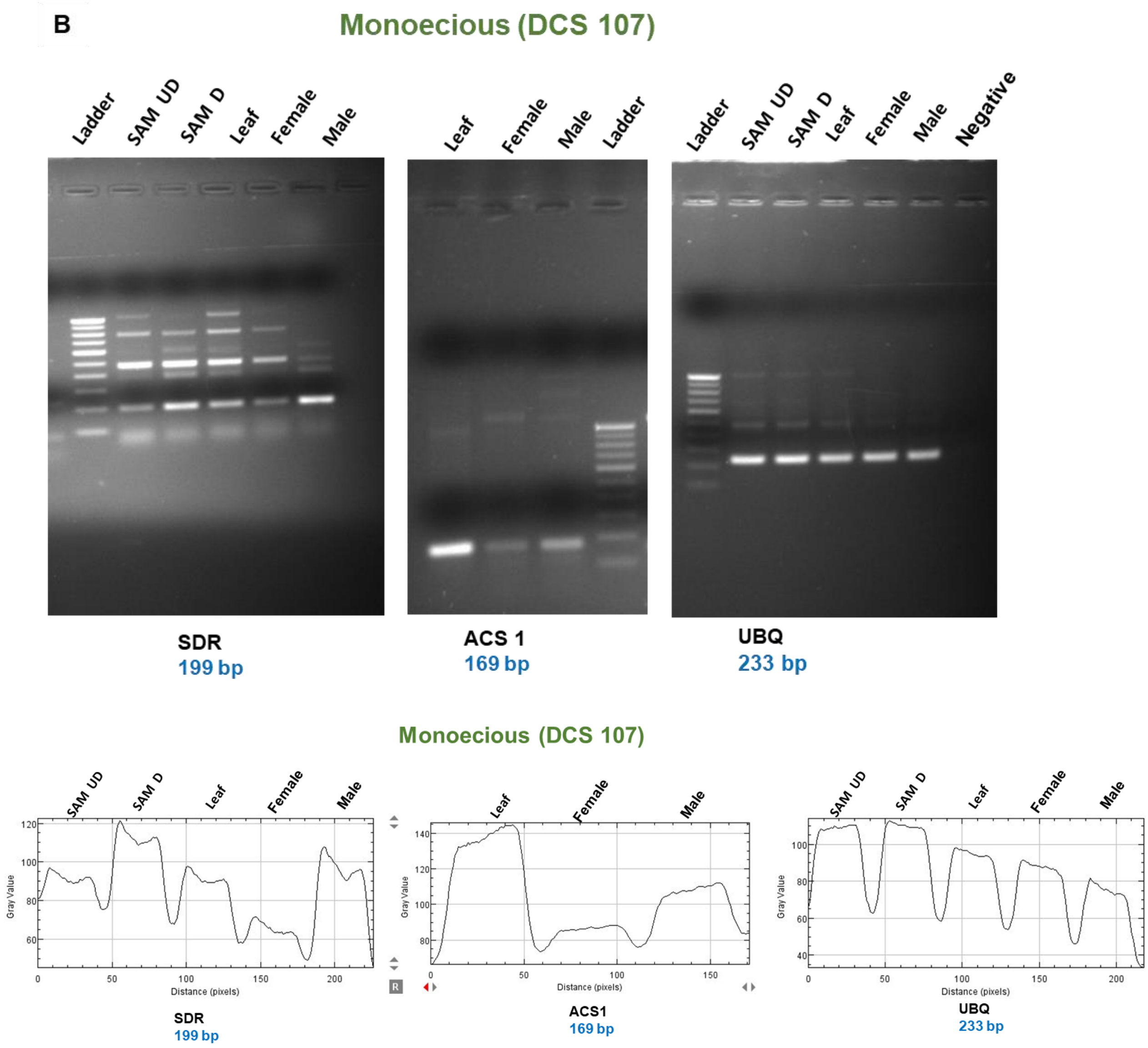
B
Monoecious (DCS 107)
Ladder
SAM UD
SAM D
Leaf
Female
Male
Leaf
Female
Male
Ladder
Ladder
SAM UD
SAM D
Leaf
Female
Male
Negative
SDR
199 bp
ACS 1
169 bp
UBQ
233 bp
Monoecious (DCS 107)
SAM UD
SAM D
Leaf
Female
Male
Gray Value
Distance (pixels)
SDR
199 bp
Leaf
Female
Male
Gray Value
Distance (pixels)
ACS1
169 bp
SAM UD
SAM D
Leaf
Female
Male
Gray Value
Distance (pixels)
UBQ
233 bp

Staminate (M 574-OS1)

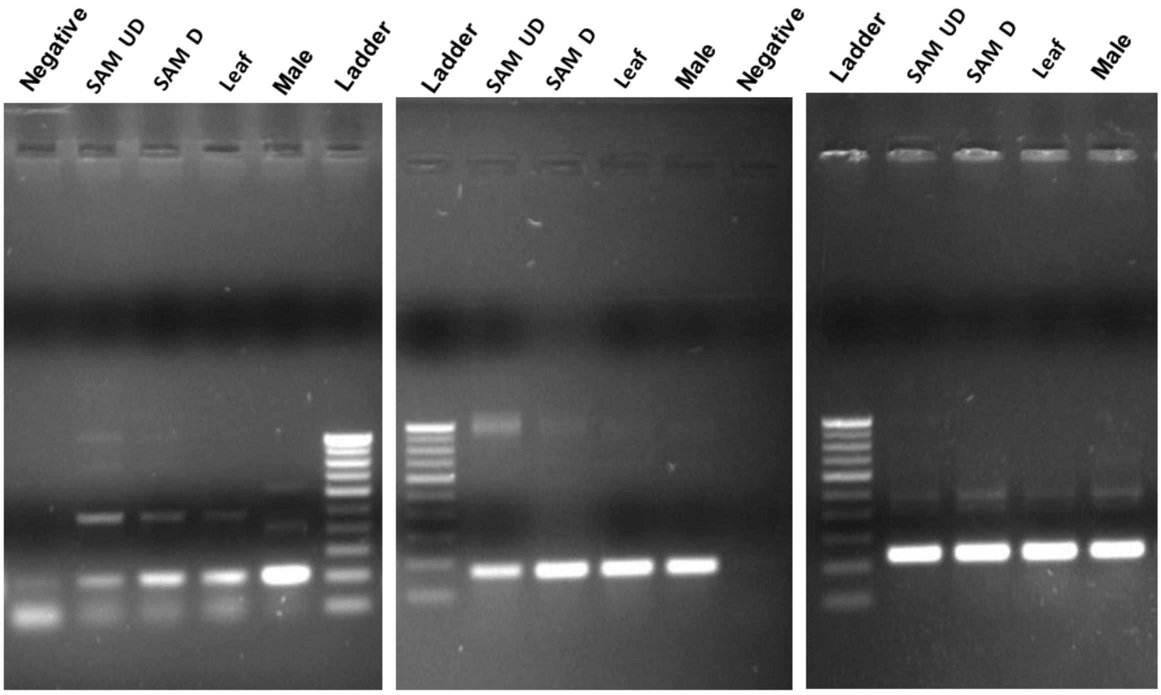

SDR
199 bp

ACS 1
169 bp

UBQ
233 bp

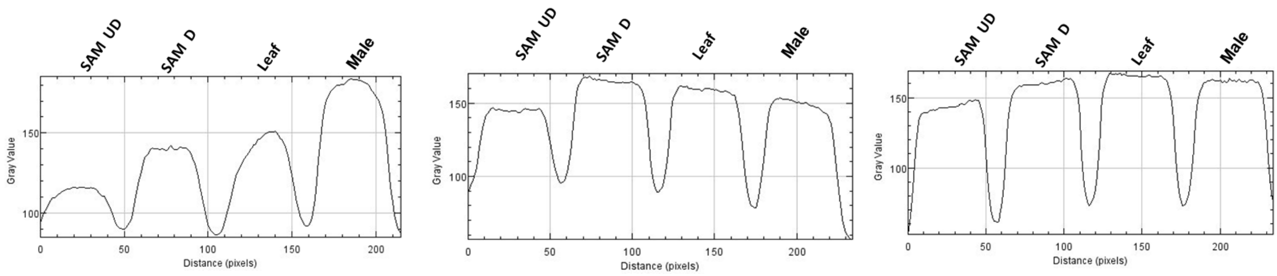

SDR
199 bp

ACS1
169 bp

UBQ
233 bp

Pistillate (DPC 9)

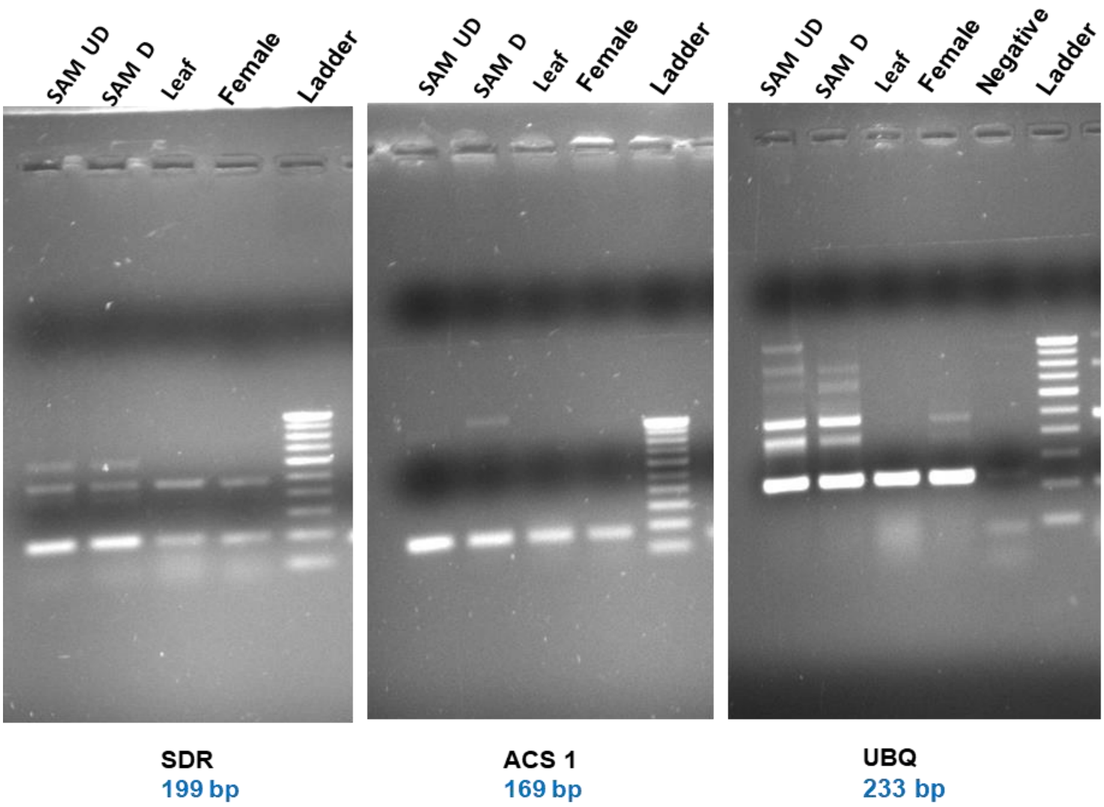

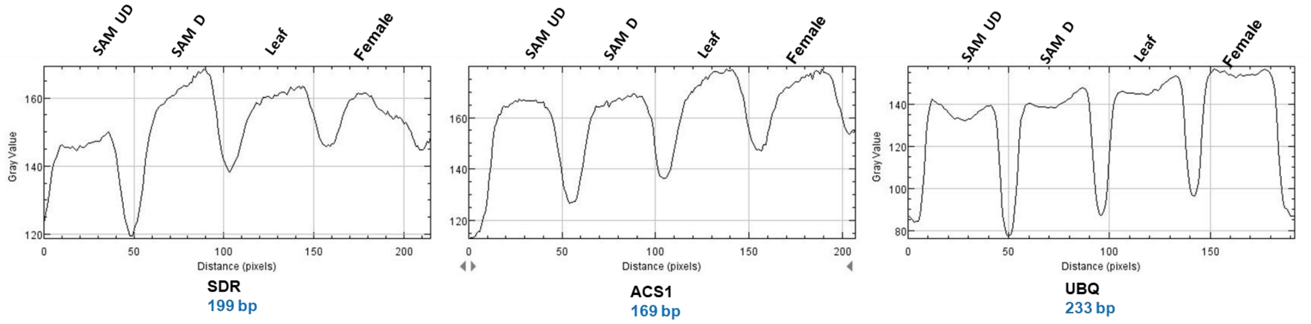

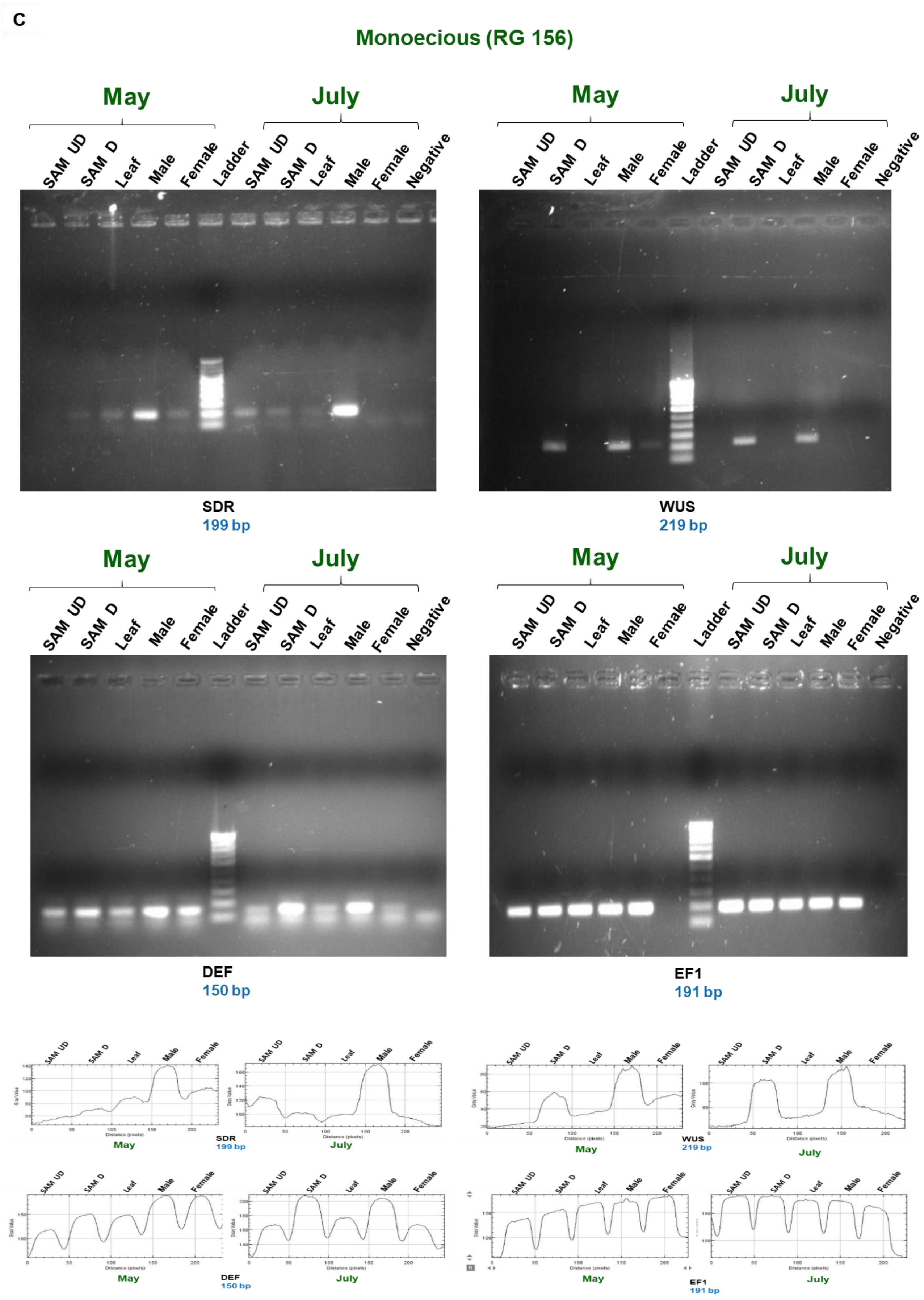

C
Monoecious (RG 156)
May
July
SAM UD
SAM D
Leaf
Male
Female
Ladder
Negative
SDR
199 bp
WUS
219 bp
DEF
150 bp
EF1
191 bp

D

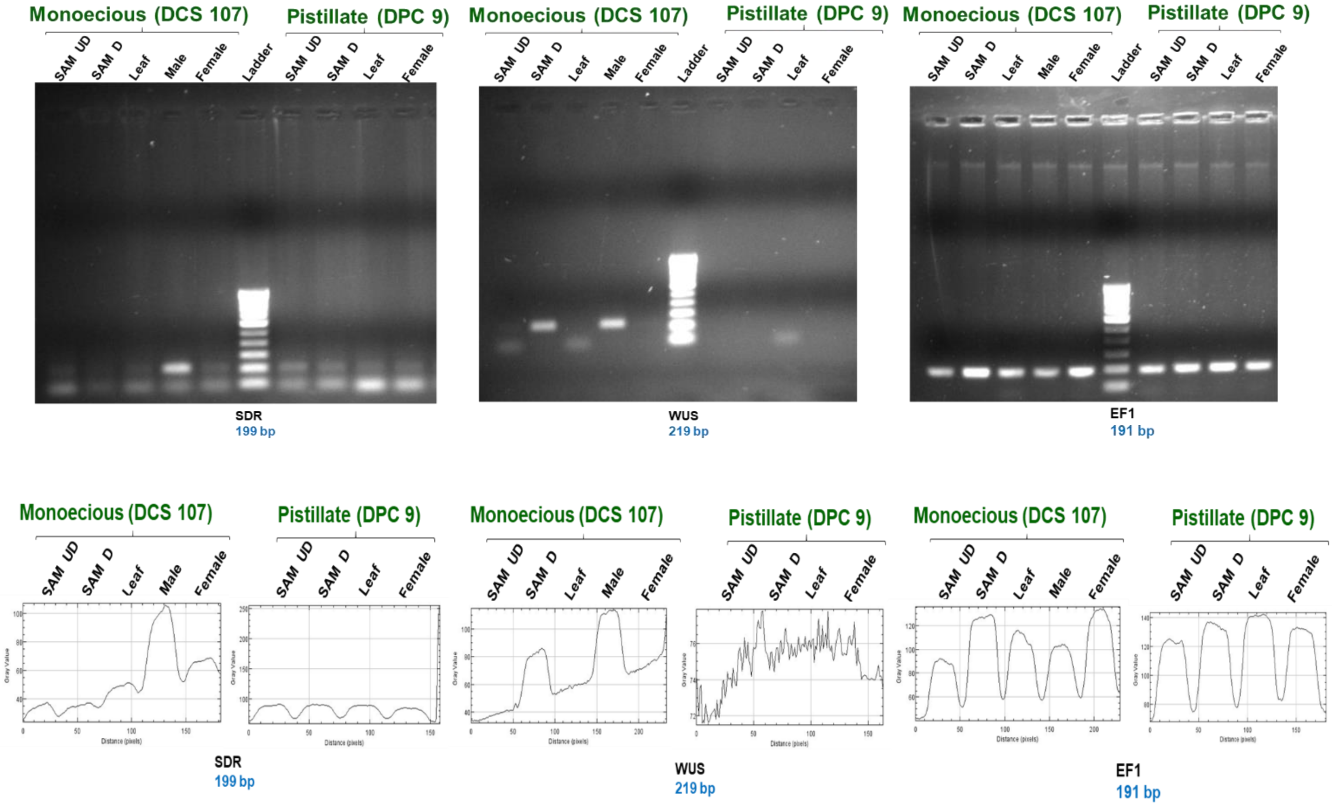
Monoecious (DCS 107)
Pistillate (DPC 9)
SAM UD
SAM D
Leaf
Male
Female
Ladder
SDR
199 bp
WUS
219 bp
EF1
191 bp
Distance (pixels)
Gray Value

E

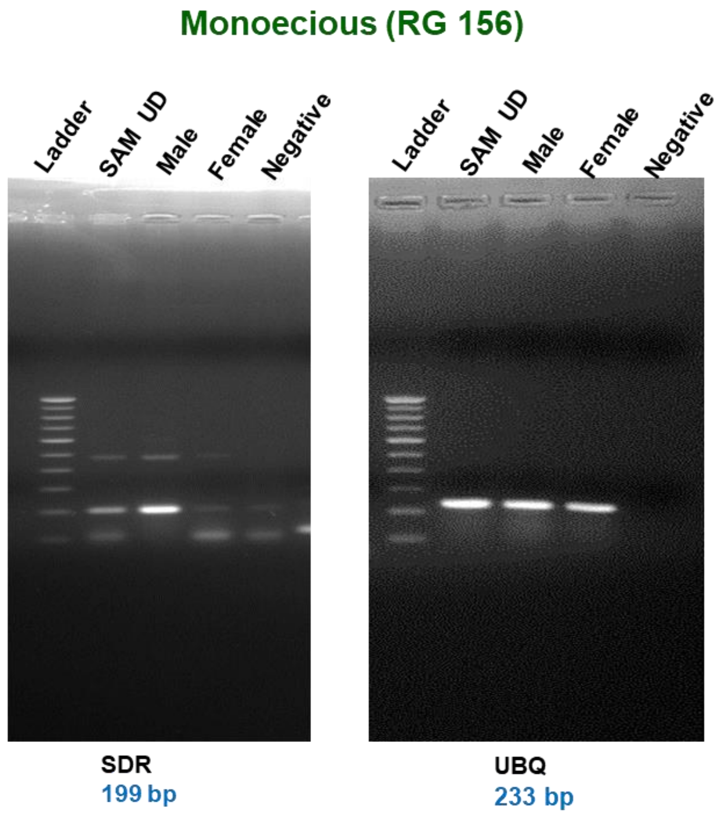
Monoecious (RG 156)
Ladder
SAM UD
Male
Female
Negative
SDR
199 bp
UBQ
233 bp

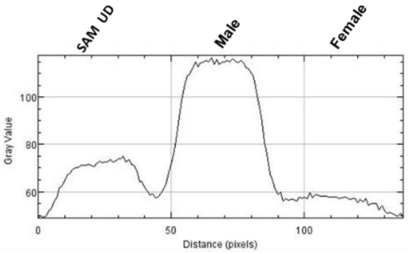

SDR
199 bp

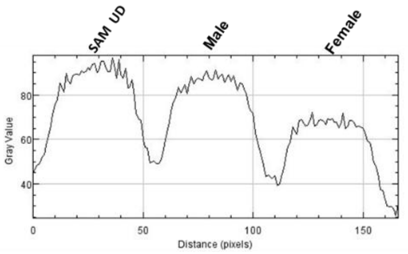

UBQ
233 bp

F

Monoecious (RG 156)

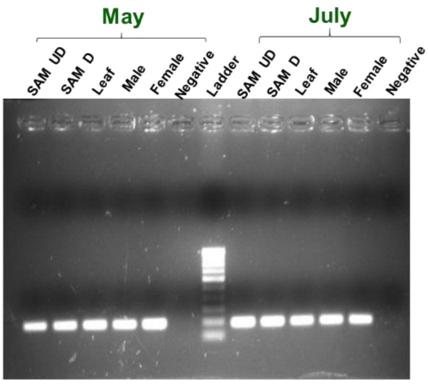

EF1
191 bp

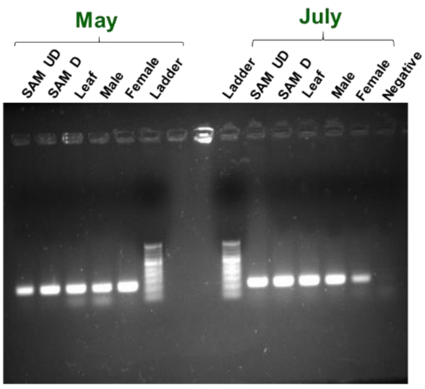

UBQ
233 bp

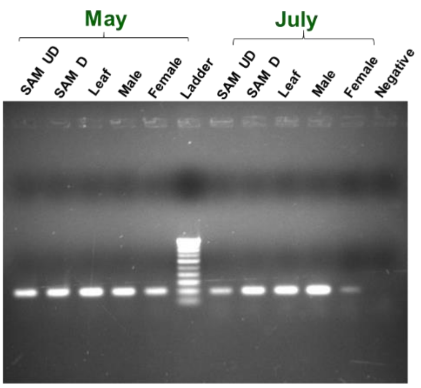

ACT
197 bp

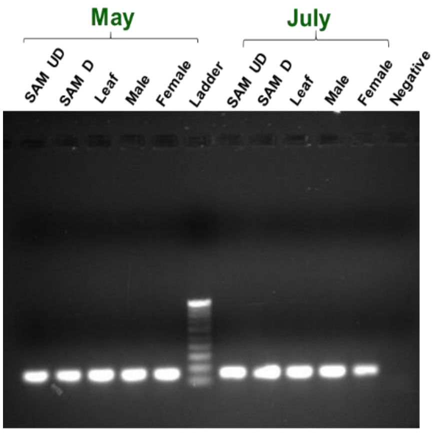

PGK
153 bp

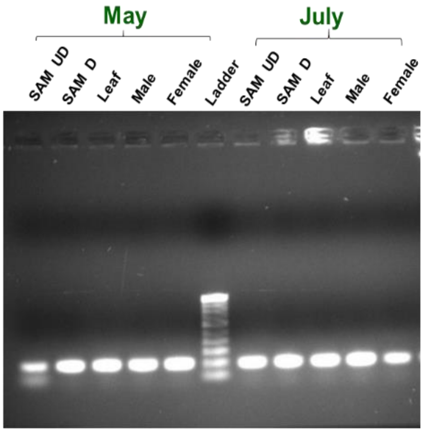

GADPH
193 bp

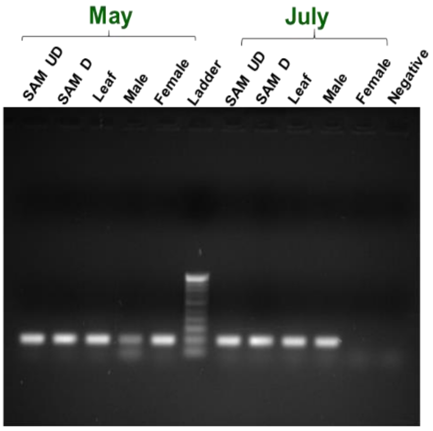

SND
190 bp

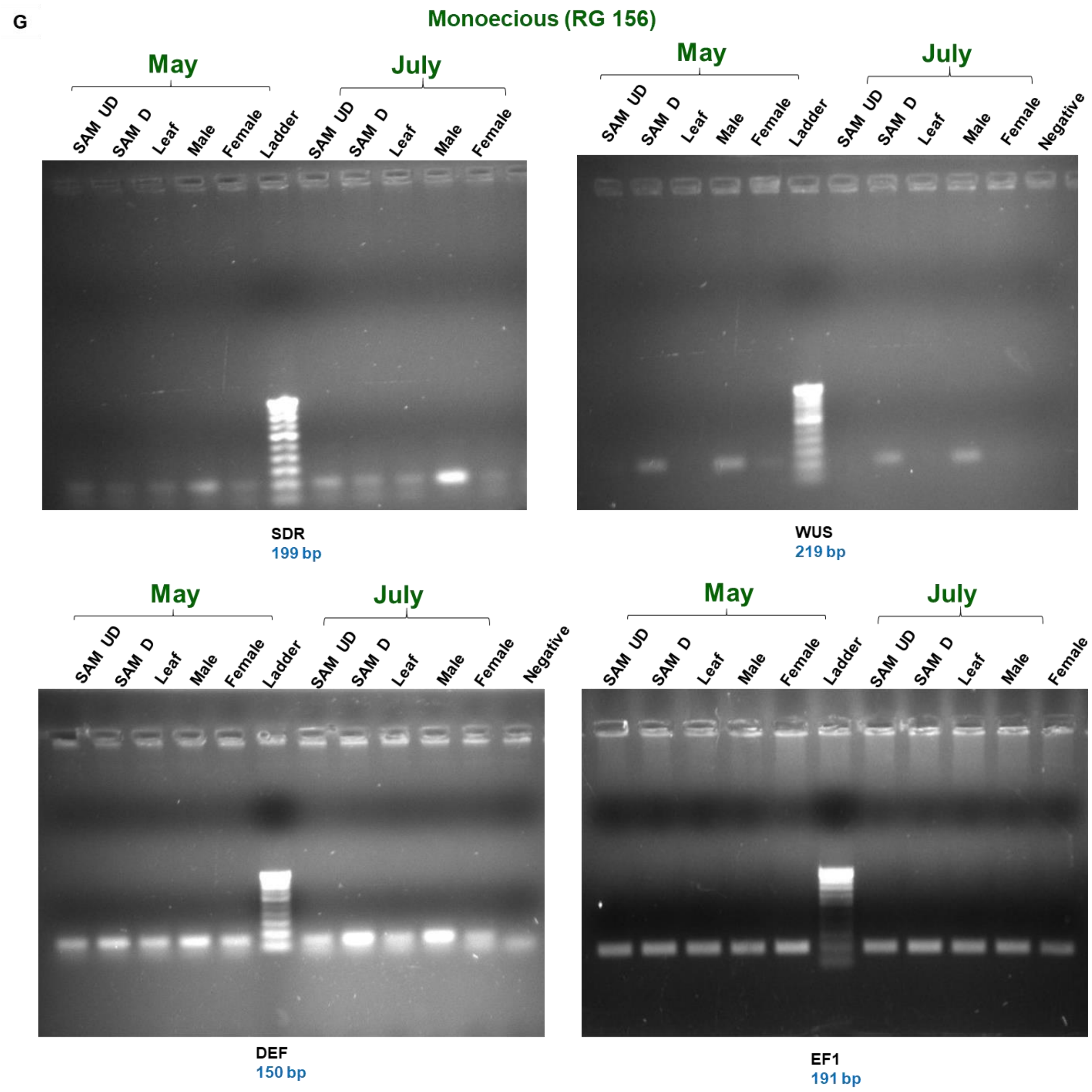

Supplementary Figure S8: Complete or whole gels showing gene expression profiling in castor genotypes by semi-quantitative RT-PCR

(**A**) Expression of 1-amino cyclopropane carboxylate synthase (*ACS* and *ACS 1*) I monoecious RG 156 **(B)** Expression of *SDR* and *ACS1* in monoecious DCS 107, staminate M 574-OS1 and Pistillate DPC 9 genotypes. (**C**) Expression of *SDR*, *DEF* and *WUS* in monoecious RG156 under two temperature conditions of May (high) and July (low). (**D**) Expression of *SDR* and *WUS* in monoecious DCS 107 and pistillate DPC 9. (**E**) Expression of *SDR* in monoecious RG 156.*UBQ* is same as that in (A). (**F**) Expression of 6 control genes in monoecious RG 156 under two temperature conditions in May and July. (**G**) Reverification of expression of *SDR*, *DEF* and *WUS* in monoecious RG 156 under different temperature conditions of May and July. *UBQ* and *EF-1* are used as internal control genes. L is 100 bp ladder, Negative is water control. Shoot apical meristem (SAM UD), differentiated SAM or inflorescence bud at stage II (SAM D), young growing leaf from inflorescence bud (leaf), young male and female buds are indicated.

The plot profiles for band intensity comparison generated using Image J is given for gels (A-E). *Non- specific amplification in one-step RT-PCR using random hexamers in (A), (B) and (E) were absent in (C), (D), (F) and (G) in two-step RT-PCR when oligo dT was used for cDNA synthesis. Experiments (A), (B) and (E) were carried out during 2015-17 and (C), (D), (F) and (G) during 2018. Samples for (A) were and (E) were collected during May 2015 and (B) collected during mid-February to 1st week of March 2017.
