# Supplementary material for: Probing the floral developmental stages, bisexuality and sex reversions in castor (Ricinus communis L.)
Source: Sci Rep. 2021 Feb 19;11:4246. doi: 10.1038/s41598-021-81781-9 (PMC7895920; doi:10.1038/s41598-021-81781-9)
Supplement: Supplementary file 3 — Supplementary Information. [file 41598_2021_81781_MOESM3_ESM.pdf]

Probing the Floral Developmental Stages, Bisexuality and Sex Reversions in Castor (*Ricinus communis* L.)

Scientific Reports

Sujatha Thankeswaran Parvathy[1*], Amala Joseph Prabakaran[2] and Thadakamalla Jayakrishna[3]

ICAR-Indian Institute of Oilseeds research, Rajendranagar, Hyderabad, Telangana, 500 030 India.

*E-mail: sujatha.parvathy@icar.gov.in, hiisuj1@gmail.com

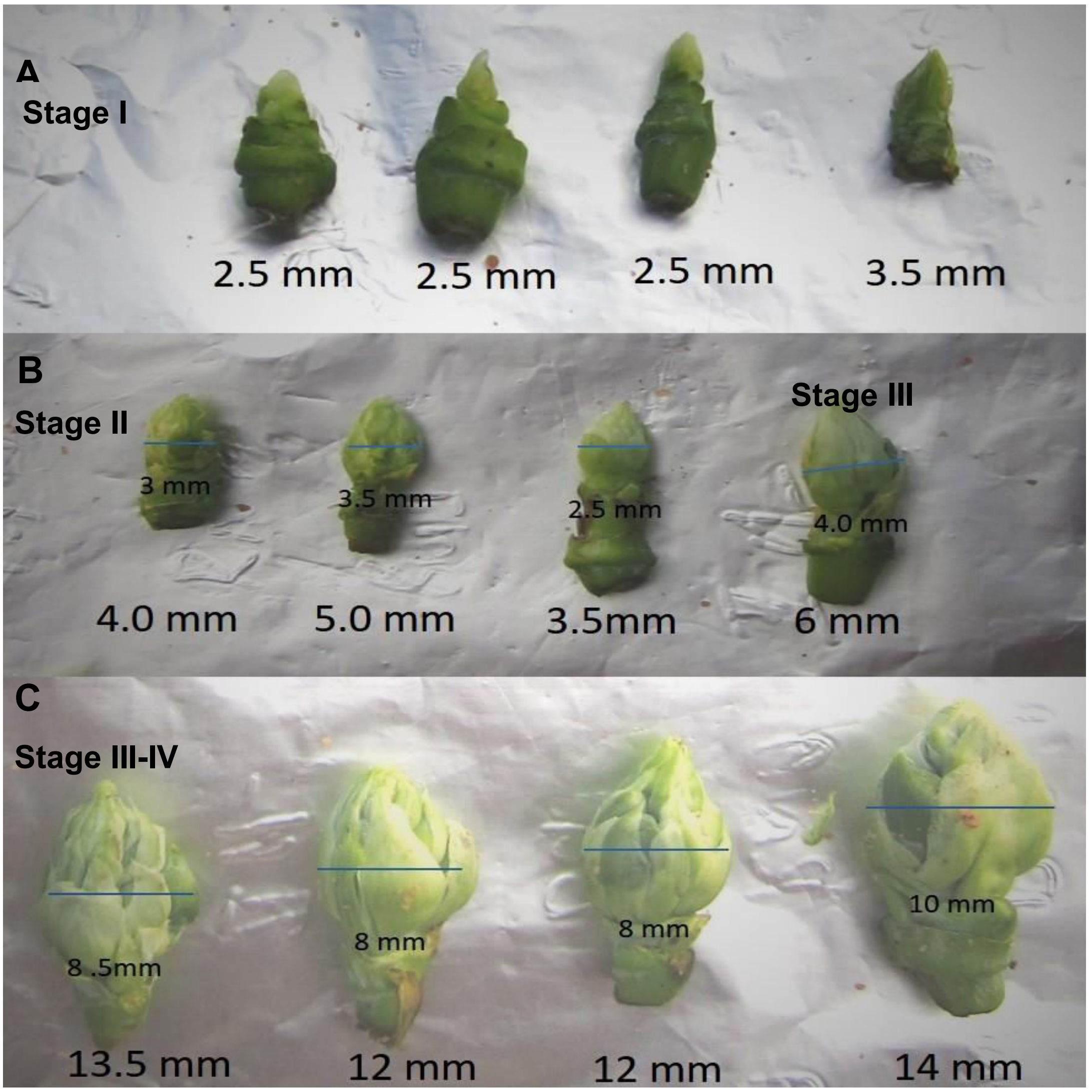

Supplementary Figure S1. Growth of inflorescence from stage I to stage IV in monoecious RG 156. (**A**) The inflorescence primordium at stage I (2.5 mm in length, 1.5-2mm in girth) not distinct externally. (**B**) Inflorescence bud at stage II (3-5 mm in length, 2.5-3.5 mm in girth) and stage III (6 mm in length and 4 mm in girth) visible externally as a bulge (**C**) Inflorescence at different stages from stage III to IV (12-14 mm length, 8-10 mm in girth). The vertical length and horizontal girth (blue line) of the primordium are indicated in mm respectively below and on the inflorescence bud.

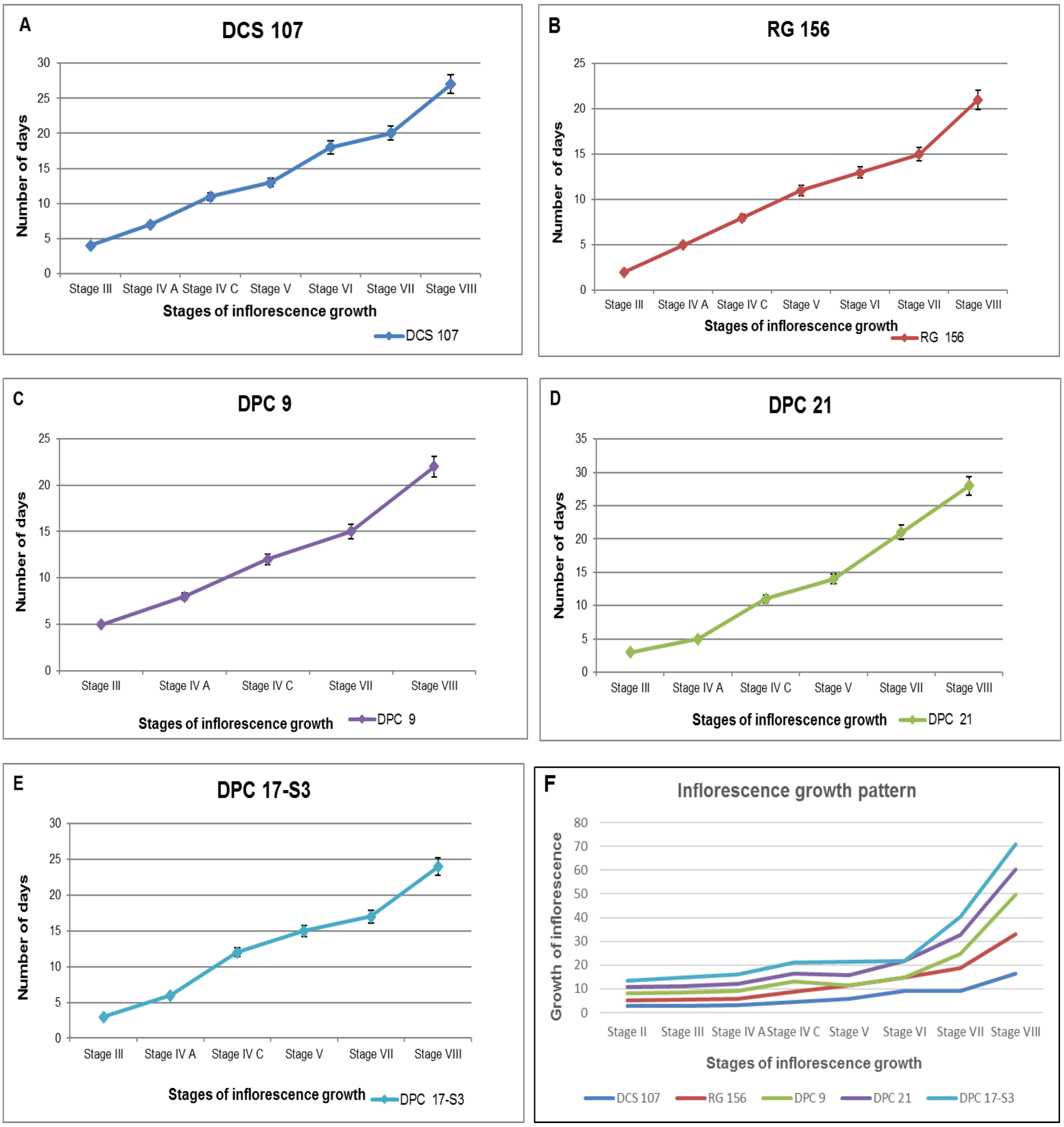
A
DCS 107
Number of days
Stage III
Stage IV A
Stage IV C
Stage V
Stage VI
Stage VII
Stage VIII
Stages of inflorescence growth
DCS 107
B
RG 156
Number of days
Stage III
Stage IV A
Stage IV C
Stage V
Stage VI
Stage VII
Stage VIII
Stages of inflorescence growth
RG 156
C
DPC 9
Number of days
Stage III
Stage IV A
Stage IV C
Stage VII
Stage VIII
Stages of inflorescence growth
DPC 9
D
DPC 21
Number of days
Stage III
Stage IV A
Stage IV C
Stage V
Stage VII
Stage VIII
Stages of inflorescence growth
DPC 21
E
DPC 17-S3
Number of days
Stage III
Stage IV A
Stage IV C
Stage V
Stage VII
Stage VIII
Stages of inflorescence growth
DPC 17-S3
F
Inflorescence growth pattern
Growth of inflorescence
Stage II
Stage III
Stage IV A
Stage IV C
Stage V
Stage VI
Stage VII
Stage VIII
Stages of inflorescence growth
DCS 107
RG 156
DPC 9
DPC 21
DPC 17-S3

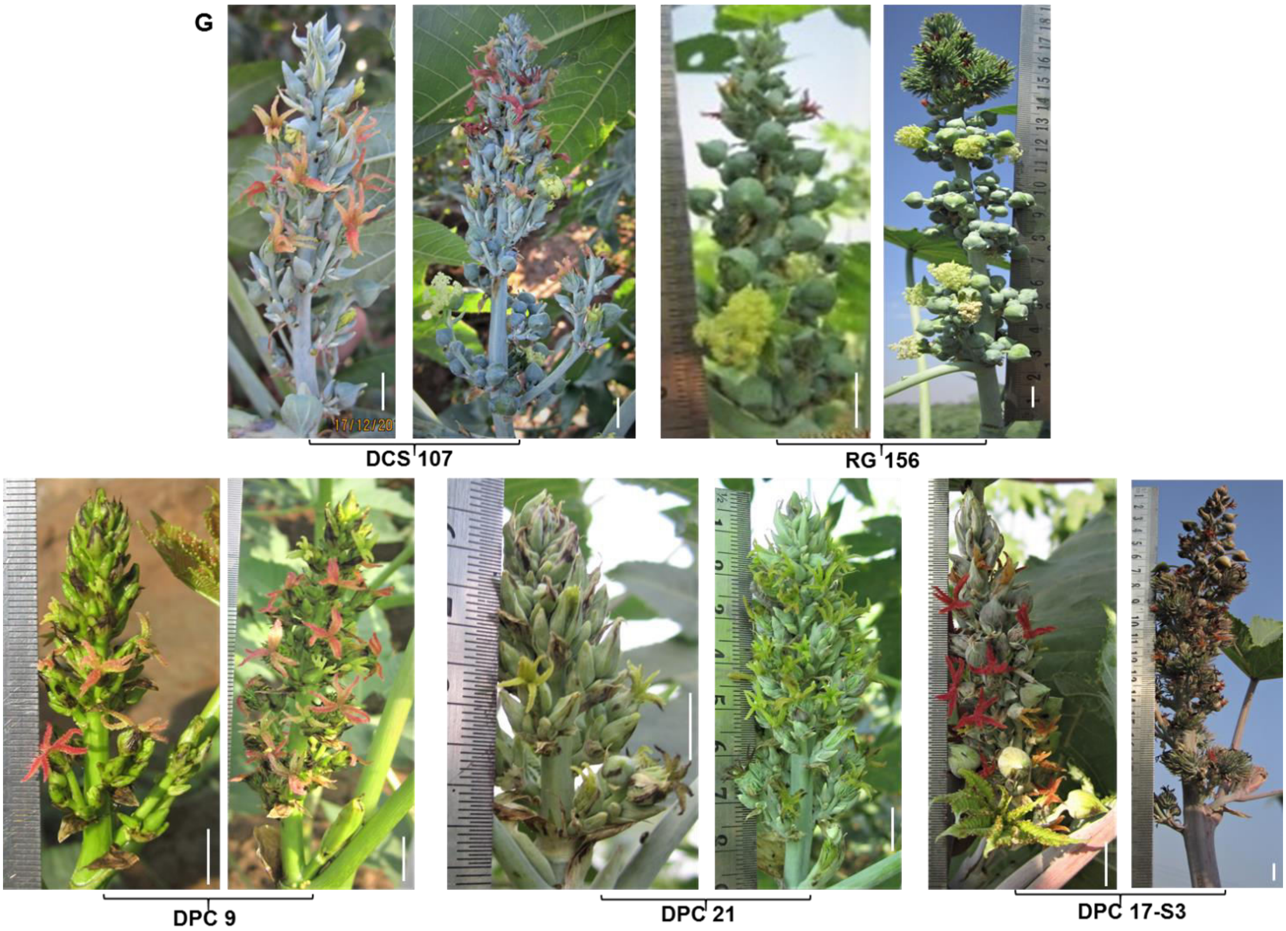

Supplementary Figure S2. Inflorescence growth stages at various days after inflorescence differentiation, growth rate and anthesis pattern in castor genotypes (**A**) DCS 107 (monoecious), (**B**) RG 156 (monoecious), (**C**) DPC 9 (pistillate), (**D**) DPC 21 (ISF) and (**E**) DPC 17-S3 (apical ISF). Stage VI (elongation) is not distinct before stage VII (anthesis) in DPC 9, DPC 21 and DPC 17-S3. The error bars at each point indicate error percentage. (**F**) Growth pattern of inflorescences indicated in time line graph (**G**) Pattern of anthesis in various castor genotypes. Scale bar: 1 cm.

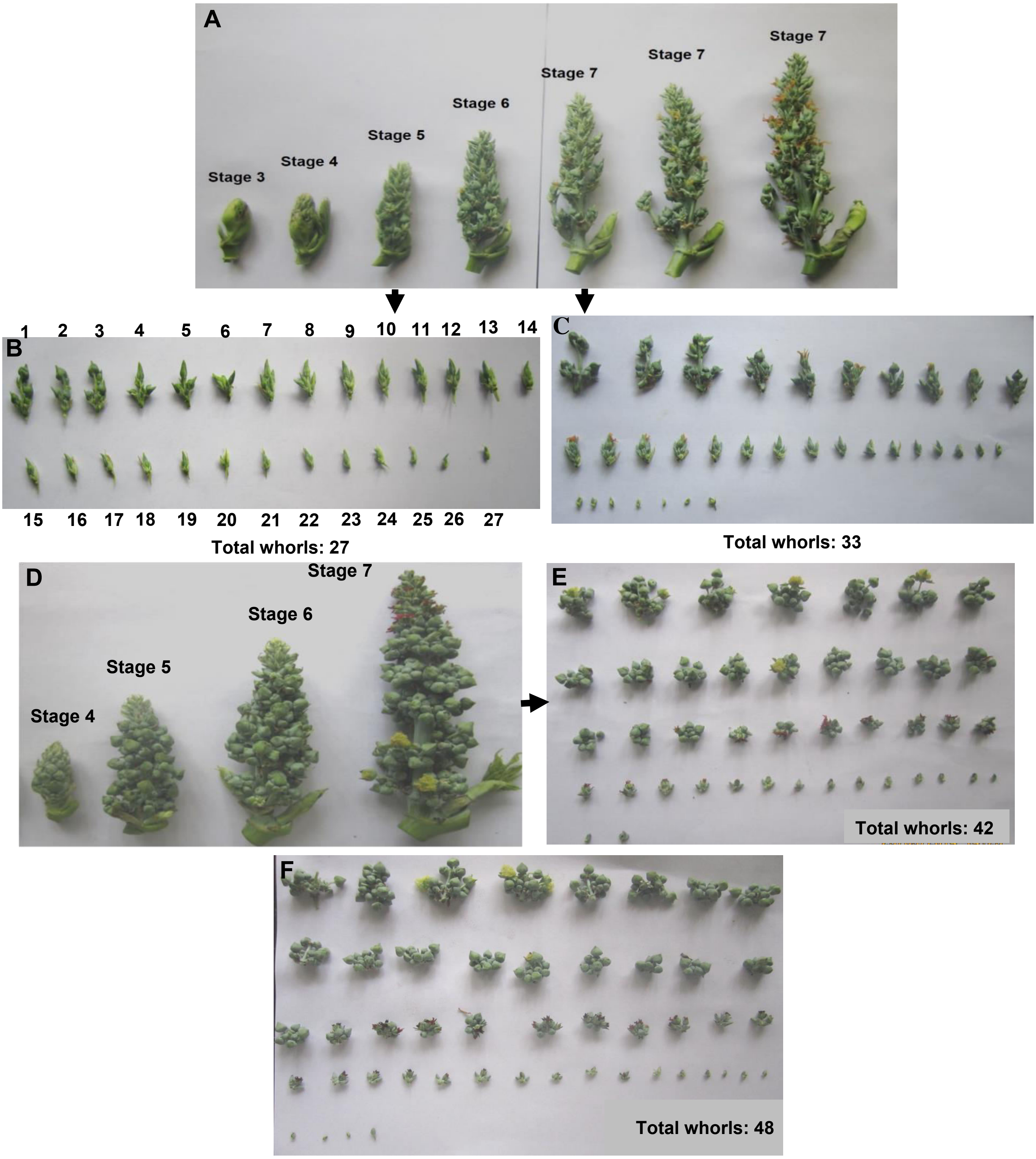
A
Stage 3
Stage 4
Stage 5
Stage 6
Stage 7
Stage 7
Stage 7
B
1 2 3 4 5 6 7 8 9 10 11 12 13 14
15 16 17 18 19 20 21 22 23 24 25 26 27
Total whorls: 27
C
Total whorls: 33
D
Stage 4
Stage 5
Stage 6
Stage 7
E
Total whorls: 42
F
Total whorls: 48

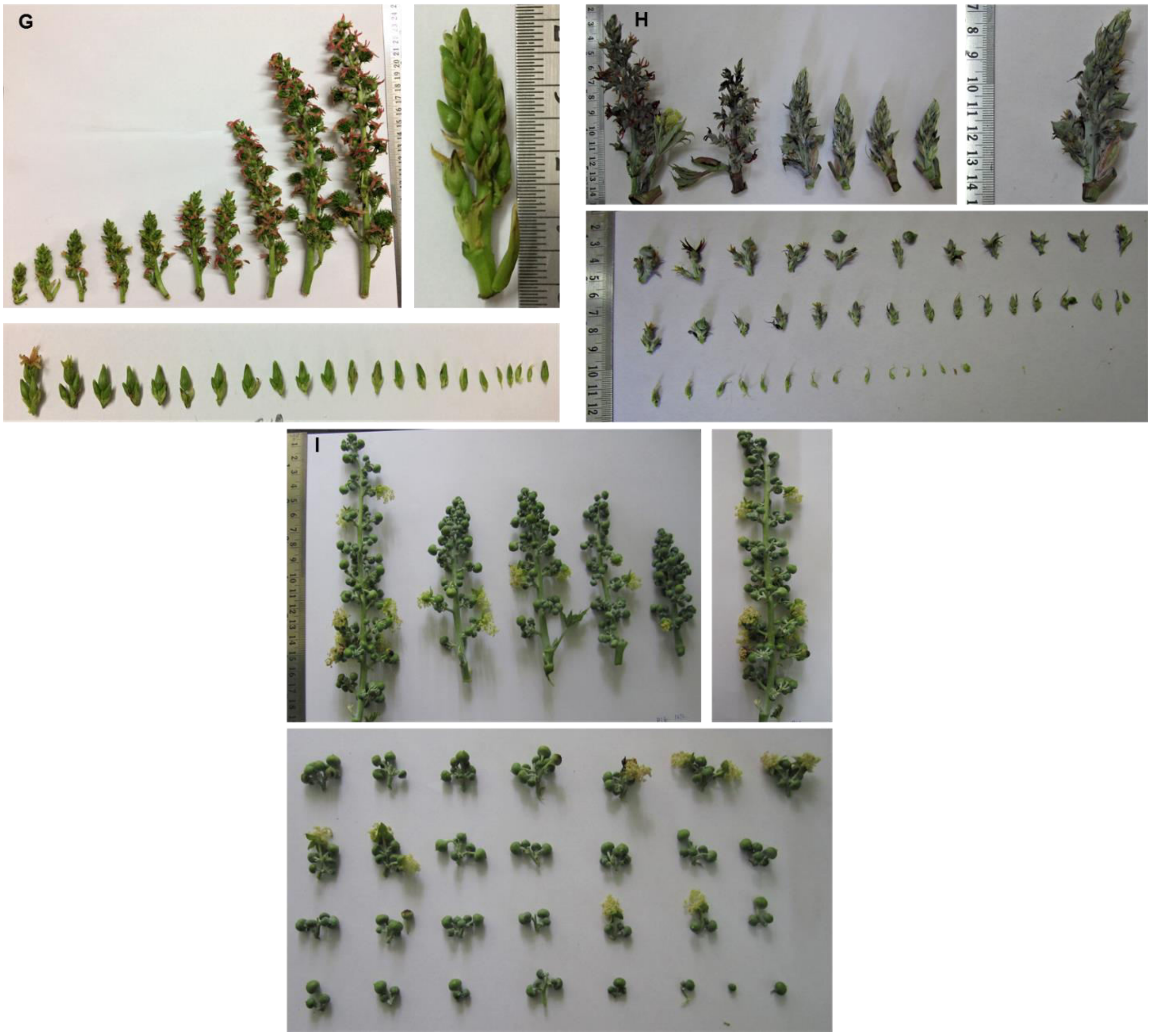

Supplementary Figure S3. Arrangement of floral whorls to elucidate architecture of inflorescences of castor genotypes. Two monoecious genotypes (**A-C**) DCS 107 and (**D-F**) RG 156 are represented. Spikes of DCS 107 (**A**) and RG 156 (**D**) at different stages of elongation and separated floral whorls from the spikes arranged I the order from bottom to top of spike for DCS 107 (**B, C**) and RG 156 (**E, F**) are shown respectively. The total number of whorls from the respective spikes (indicated by arrow) are shown below. Inflorescence architecture of (**G**) DPC 9 (**H**) DPC17-S3 and (**I**) M 574-OS1 were similarly elucidated.

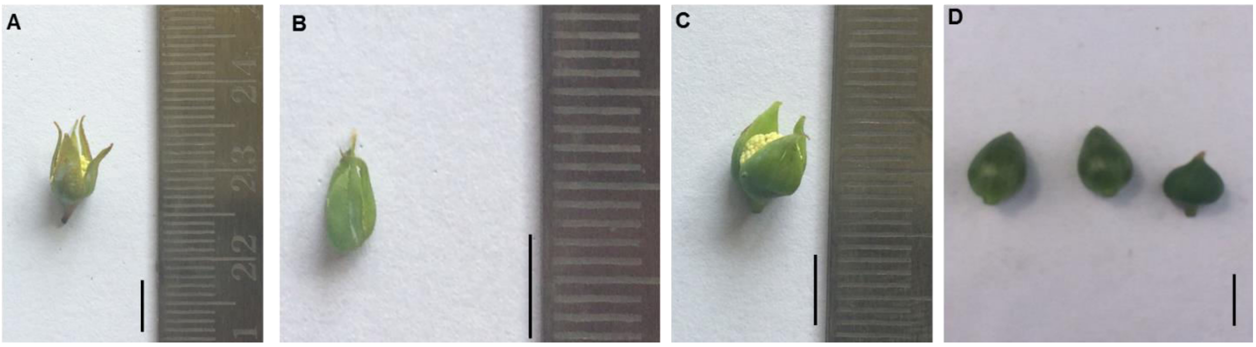

Supplementary Figure S4. Abnormal shape in castor flower buds

(**A, B**) Elongated male flower buds (**C**) Normal round (spherical) male bud (**D**) Nearly-round female flower buds adjacent to normal male bud (right extreme). Graduations of 30 cm metal scale is shown at side. Scale bar= 50 mm

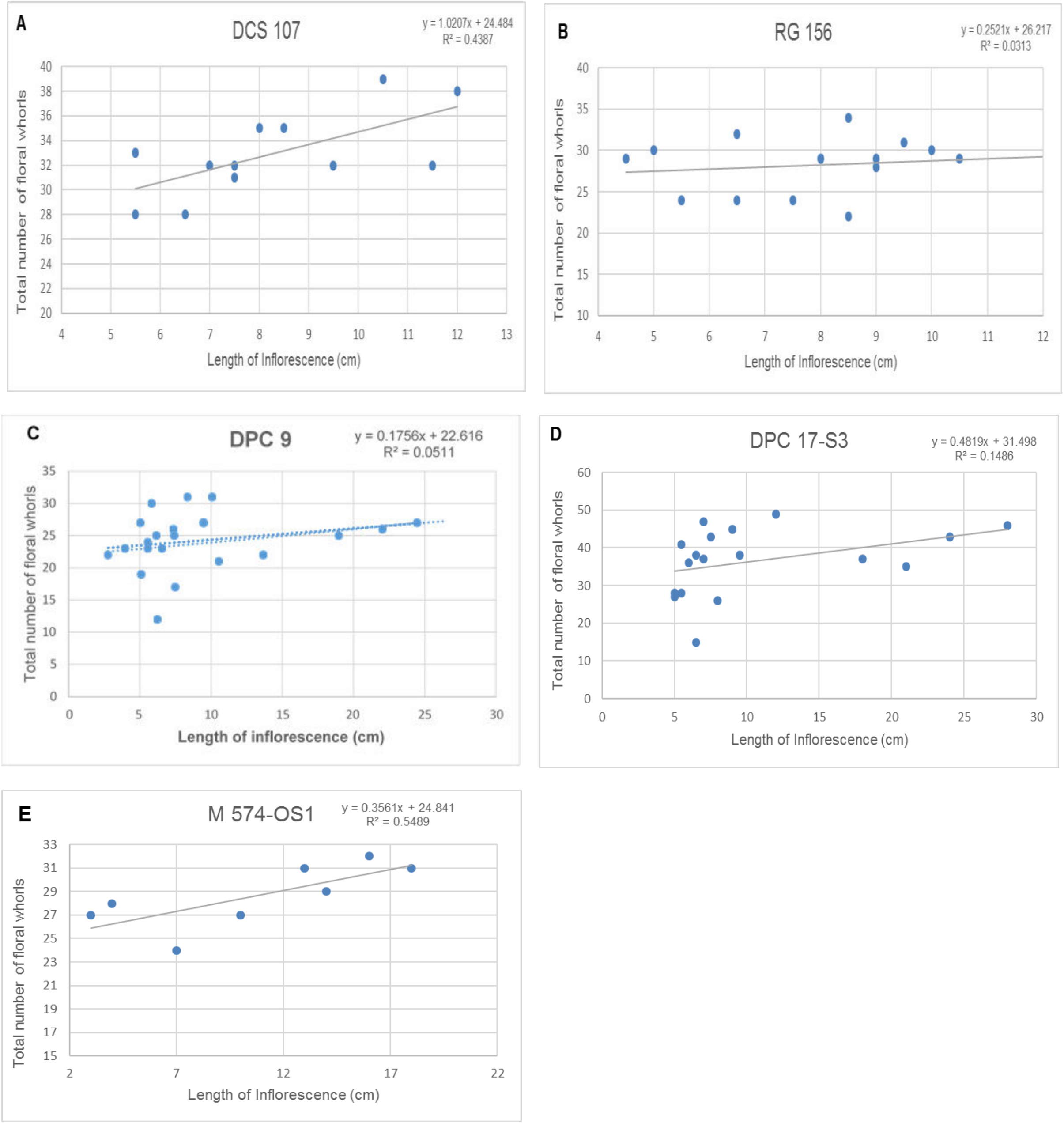

Supplementary Figure S5. Scatter plot showing the relation total number of floral whorls along with elongation of inflorescence after emergence in castor genotypes (**A**) DCS 107 (**B**) RG 156 (**C**) DPC 9 (**D**) DPC 17-S3 (**E**) M574-OS1.The regression equation is shown on top right of the scatter plot diagram.

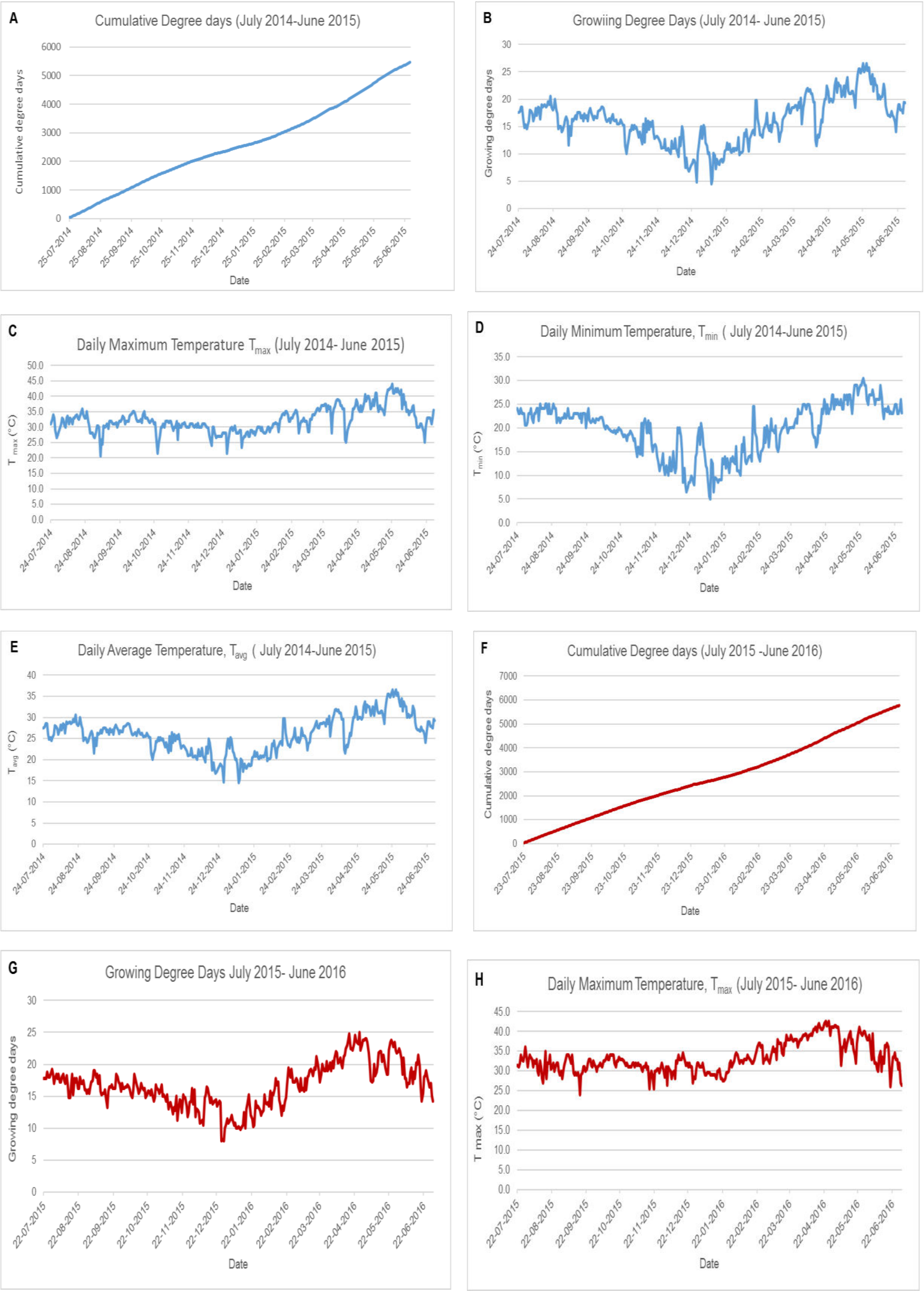

A
Cumulative Degree days (July 2014-June 2015)
Cumulative degree days
Date
B
Growiing Degree Days (July 2014- June 2015)
Growing degree days
Date
C
Daily Maximum Temperature T_{max} (July 2014- June 2015)
T_{max} (°C)
Date
D
Daily Minimum Temperature, T_{min} (July 2014-June 2015)
T_{min} (°C)
Date
E
Daily Average Temperature, T_{avg} (July 2014-June 2015)
T_{avg} (°C)
Date
F
Cumulative Degree days (July 2015 -June 2016)
Cumulative degree days
Date
G
Growing Degree Days July 2015- June 2016
Growing degree days
Date
H
Daily Maximum Temperature, T_{max} (July 2015- June 2016)
T max (° C)
Date

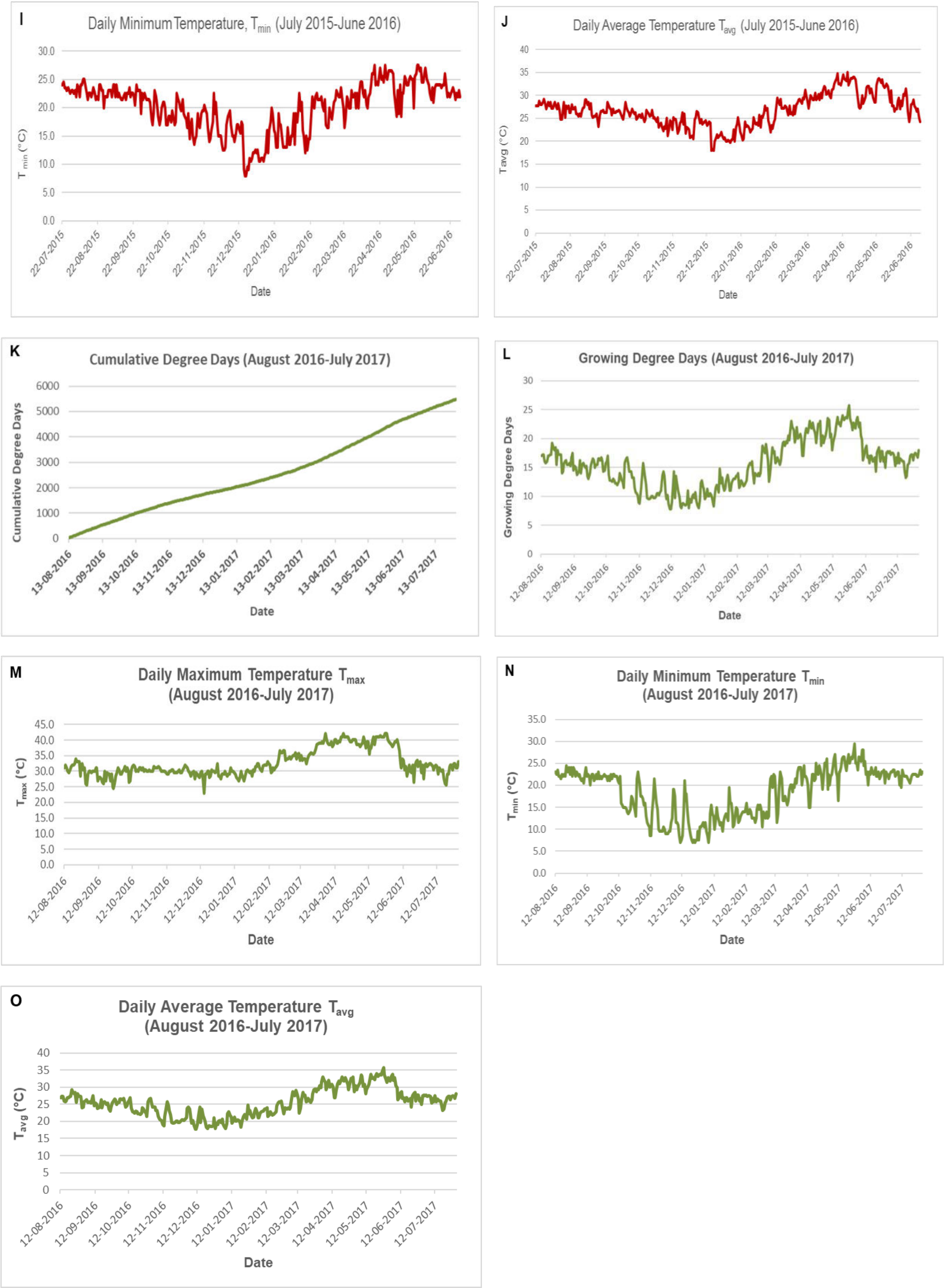

Supplementary Figure S6. Weather parameters Cumulative degree days (CDD), Growing Degree Days (GDD), daily maximum (T_{max}), minimum (T_{min}) and average (T_{avg}) temperatures for the cropping season (**A-E**) 2014-15, (**F-J**) 2015-16 and (**K-O**) 2016-17.

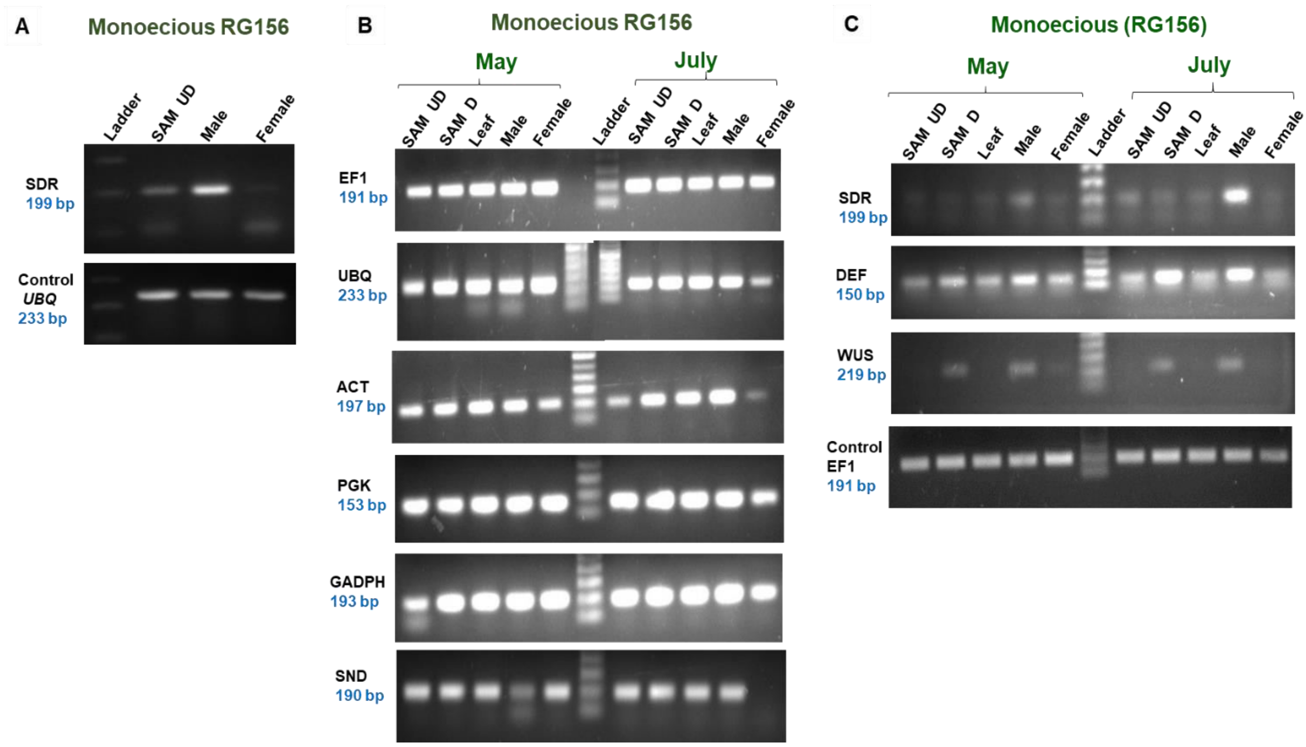

Supplementary Figure S7. Expression of genes in various tissues in castor genotypes verified by semi-quantitative RT-PCR. (**A**) Expression of *SDR* in Monoecious RG 156 using one-step RT-PCR (**B**) Standardisation of various control genes at two different temperature conditions during May (38-42°C) and July (25.5-33°C) in RG 156 using two-step RT-PCR (**C**) Expression of candidate genes in RG 156 during May and July re-confirmed by two step RT-PCR. *UBQ* and *EF-1* are used as internal control genes. Ladder is 100 bp ladder. Samples from vegetative undifferentiated Shoot apical meristem (SAM UD), differentiated SAM (SAM D), young growing leaf from inflorescence bud (leaf), young male and female buds are indicated.

Probing the Floral Developmental Stages, Bisexuality and Sex Reversions in Castor (*Ricinus communis* L.)

Scientific Reports

Sujatha Thankeswaran Parvathy[1*], Amala Joseph Prabakaran[2] and Thadakamalla Jayakrishna[3]

ICAR-Indian Institute of Oilseeds research, Rajendranagar, Hyderabad, Telangana, 500 030 India.

*E-mail: sujatha.parvathy@icar.gov.in, hiisuj1@gmail.com

Supplementary Table S1. Morphological stages of inflorescence development in castor: The stages after differentiation of apical meristem into reproductive/ inflorescence meristem are described.

Stages	Description
**Stage I**	Inflorescence primordia is differentiated but not prominent nor visible
**Stage II**	Inflorescence primordia is prominent, visible and hard to touch
**Stage III**	Inflorescence is covered by bracts and not emerged, increased girth of spike, Flower buds fully formed, not visible outside
**Stage IV**	A. Inflorescence almost covered by bracts starts emerging out, marked by opening of bracts from tip
	B. Inflorescence emerging out and partially covered by bracts (half or 50% open)
	C. Inflorescence emerging out and 3/4th or 75% open.
**Stage V**	Inflorescence has emerged completely or fully open, individual flower buds remain unopened.
**Stage VI**	Elongation of fully emerged inflorescence occurs, but flower buds remain unopened.
**Stage VII**	Anthesis or Flower bud opening
**Stage VIII**	Capsule formation (after pollination) and capsule maturation

Supplementary Table S2. Stage transition and growth of inflorescence in different castor genotypes

A. Number of days taken for transition to each stage of development of inflorescence.

Genotype	Stage II-III	Stage III-IV A	Stage IV A-IV C	Stage IV C-VII	Stage VII-VIII
DCS 107	4 ± 0.3	3 ± 0.63	4 ± 0.58	5 ± 0.2	7 ± 0.25
RG 156	2 ± 0.33	3	3	7 ± 0.14	6 ± 0.33
DPC 9	5 ± 0.88	3 ± 0.48	4	3	7 ± 0.34
DPC 21	3 ± 0.4	2 ± 0.24	6	10	7
DPC 17-S3	3	3	6	6	7

Mean of observations adjusted to nearest whole number ± $SE_{(mean)}$are shown.

B. Stage of inflorescence development at different days from stage II.

Developmental stage of inflorescence at nth day							
**Genotype**	**Stage III**	**Stage IVA**	**Stage IVC**	**Stage V**	**Stage VI**	**Stage VII**	**Stage VIII**
DCS 107	4	7	11	13	18	20	27
RG 156	2	5	8	11	13	15	21
DPC 9	5	8	12	*	*	15	22
DPC 21	3	5	11	14	*	21	28
DPC 17-S3	3	6	12	15	*	17	24

*'indicates no observations are available since stage transitions are not distinct. The total number of days from stage II are shown.

C. Inflorescence growth (cm) of castor genotypes at different stages

Genotype	Stage II	Stage III	Stage IV A	Stage IV C	Stage V	Stage VI	Stage VII	Stage VIII
DCS 107	2.86 ± 0.11	2.89 ± 0.16	3.2 ± 0.12	4.43 ± 0.20	5.88 ± 0.69	9 ± 1	9.214 ± 0.67	16.5 ± 1.3
RG 156	2.17 ± 0.33	2.5 ± 0.29	2.5 ± 0.29	4.25 ± 0.22	5.58 ± 0.20	5.75 ± 0.25	9.583 ± 0.65	16.65 ± 0.58
DPC 9	3 ± 0.29	3 ± 0.20	3.5 ± 0.18	4.3 ± 0.4	*	*	6 ± 0.69	16.41± 0.71
DPC 21	2.67 ± 0.17	2.81 ± 0.09	3 ± 0.16	3.45 ± 0.13	4.25 ± 0.26	7 ± 1	7.833 ± 0.88	10.75 ± 1.5
DPC 17-S3	2.8 ± 0.17	3.5 ± 0.20	3.75 ± 0.14	4.5 ± 0.3	5.8 ± 0.6	*	7.8 ± 0.53	10.5 ± 0.39

The values represent mean of observations from different spikes.'*'indicates no observations are available since stage transitions are not distinct.

D. Elongation (cm)of inflorescence after capsule setting

Genotype	Stage VIII	Stage VIII A	Stage VIII B
DCS 107	16.5 ± 1.3	19 ± 0.53	24.5 ±
DPC 9	16.42 ± 0.71	21.72 ± 0.9	28.28 ± 1.36

Stage I: Inflorescence primordia initiation, stage II: inflorescence bud visible, stage III Growth of inflorescence bud Stage IV of inflorescence opening with 3 sub-stages IVA beginning of inflorescence opening IVC: inflorescence 3/4th opened Stage V: Complete spike emergence Stage VI: Spike elongation Stage VII: Anthesis. Stage VIII is capsule formation stage where capsules are immature, VIII A is inflorescence elongation after stage 8 where capsules mature, and stage VIII B is of fully grown inflorescence with mature capsules.

Supplementary Table S3 A. Floral architecture of inflorescences in various castor genotypes with distinct sex expression phenotypes.

Sl no	Inflorescence length (cm)	Total number of whorls	Whorl of female flower appearance	Completely female whorl*
**DCS 107**				
1	5.5	28	2	10
2	5.5	33	1	10
3	6.5	28	1	17
4	7	32	1	2,4
5	7	32	3	9, 11
6	7.5	32	1	10
7	7.5	31	1,3	8
8	8	35	1	12
9	8.5	35	1	11,12, 15,16,18,20-27,29
10	9.5	32	3	9
11	10.5	39	3	19
12	11.5	32	1	17
13	12	38	1	23
	**r=0.662**			
**DCS 107 Secondary**				
1	6.2	27	1	7
2	8.5	33	1	10
3	10.8	33	1	11
4	11.5	34	1	8
5	13	34	1	17
6	17	34	1	10
	**r=0.741**			
**RG 156**				
1	4.5	29	15	15
2	5	30	16	16
3	5.5	24	13	16
4	6.5	24	15	15
5	6.5	32	19	21
6	7.5	24	14	15
7	8	29	17	21
8	8.5	22	11	15
9	8.5	34	19	23
10	9	29	15	18
11	9	28	16	18
12	9.5	31	16,18	19
13	10	30	17	22
14	10.5	29	17	18

15	13.5	29	16.17	19
	**r=0.177**			
**RG 156 Secondary**				
1	10.5	44	18	30
2	13.5	55	22	29
3	17	36	24	31
4	17.4	55	15	23
5	17.5	46	20	31
6	18	56	10	23
	**r=0.138**			
**DPC 9**				
**Sl. no**	**Inflorescence Length (cm)**	**Total number of whorls**	**Whorls of female flower appearance**	**Whorl with male flowers**
1	2.8	22	1	Nil
2	4	23	1	Nil
3	5	27	1	Nil
4	5.1	19	1	Nil
5	5.6	23	1	Nil
6	5.6	24	1	Nil
7	5.8	30	1	Nil
8	6.1	25	1	Nil
9	6.2	12	1	Nil
10	6.5	23	1	Nil
11	7.3	26	1	Nil
12	7.4	25	1	Nil
13	7.5	17	1	Nil
14	8.4	31	1	Nil
15	9.5	27	1	Nil
16	9.5	27	1	Nil
17	10	31	1	Nil
18	10.5	21	1	Nil
19	13.6	22	1	Nil
20	19	25	1	Nil
21	22	26	1	Nil
22	24.5	27	1	Nil
	**r=0.225**			
**Sl no.**	**Inflorescence Length (cm)**	**Total number of whorls**	**Whorl of female flower appearance**	**Whorls with male flowers**
**DPC 17-S3**				
1	5.5	41	6	1,2,3,4,5,
2	5	28	1	nil

3	5	27	1	5,7,8
4	5.5	28	1	5,6,7,8, 10
5	6	36	1	1-5, 7-8, 10-26, 30-33
6	6.5	15	1	4,5,6,8
7	6.5	38	1	2-4, 12, 15-18, 20,21,26, 30-32
8	7	37	1	5,14,20,23,26,29
9	7	47	1	1,4,17-19, 22, 28, 30, 32
10	7.5	43	1	1,3,5, 13, 14, 23-41
11	8	26	1	14-15, 19-22, 24, 26
12	9	45	1	1,3,5,9,11,13,21,23,25-27
13	9.5	38	1	5-6, 14, 27-28, 33-35
14	12	49	1	all whorls
15	18	37	1	20,22-23, 29, 33-34
16	21	35	1	1-18, 24, 30
17	24	43	1	5, 7, 10-11, 13-40
18	28	46	1	3-7, 11-13, 18-21, 23-31, 34-44
	**r=0.385**			
**Sl no**	**Inflorescence length (cm)**	**Total number of whorls**	**Whorl of male flower appearance**	**Completely male whorl***
**M574-OS1**				
1	3	27	1	1
2	4	28	1	1
3	7	24	1	1
4	10	27	1	1
5	13	31	1	1
6	14	29	1	1
7	16	32	1	1
8	18	31	1	1
	**r=0.741**			

*Completely female whorl with single value denotes from that whorl onwards. The values represented are for inflorescences of tertiary or quaternary orders unless specified. The Pearson's correlation coefficient (r) for length of inflorescence and total number of floral whorls are indicated separately for each genotype.

B. Test of significance of correlation coefficient between inflorescence length and number of floral whorls in castor genotypes.

Genotype	Degrees of freedom df (n-2)	One tailed t-test df=(n-2)		Test of significance for Coefficient of correlation, r)		p value in regression	
		Calculated t (t_{cal})	Table value ($t_{critical}$) P=0.05	Calculated r	Table value ($r_{critical}$) at α=0.05 level of significance		
DCS 107	11	2.929	1.796	0.662	0.476	0.013	$p < 0.05$
RG 156	13	0.648	1.771	0.177	0.441	0.528	$p > 0.05$
DPC 9	20	1.033	1.725	0.225	0.36	0.312	$p > 0.05$
DPC 17-S3	16	1.669	1.746	0.385	0.4	0.114	$p>0.05$
M 574-OS1	6	2.703	1.943	0.741	0.621	0.035	$p < 0.05$
Secondary branch orders							
DCS 107	4	2.207	2.132	0.741	0.729	0.092	$p > 0.05$
RG 156	4	0.279	2.132	0.138	0.729	0.794	$p > 0.05$

Supplementary Table S4. Bisexuality in castor flowers A. Occurrence of bisexuality in castor during summer

Genotype	Phenotype (normal growing season)	Phenotype (summer 2nd and 3rd week of May)	Occurrence of bisexual flowers
DCS 107	Monoecious<br>3-4 whorls (30-50%) male flowers at bottom	90% male throughout inflorescence, Interspersed Staminate Flowers (ISF)	Yes<br>Terminal
RG 156	Monoecious<br>70-80% male flowers alone at bottom	90-95% male flowers, Tip flowers male occasionally, female flower buds round	Yes<br>Female flowers with reverted stamens
DPC 9	Completely pistillate	Interspersed staminate flowers (ISF)	Yes<br>Reverted female flowers with rudimentary stamens
DPC 16	Completely pistillate 3-5% male flowers at random positions predominant towards base of inflorescences	90-95% male flowers	Yes<br>Subterminal
DPC 21	Emerges as completely pistillate with ISF at capsule formation	80-85 % male throughout the inflorescence	Yes<br>Subterminal and random
DPC 17-S3	Apically interspersed staminate	90-95% male flowers	Yes<br>Terminal and subterminal
M 574-OS1	Staminate inflorescence (4th order and above), tip bisexual flower	Completely staminate	Yes<br>Terminal and subterminal
DPC 9-OS2	Predominantly staminate with tip bisexual flower	Completely staminate	Yes<br>Terminal and subterminal

B. Bisexuality observed in flowers collected at random (Observations were taken during July 2017)

Sl No	Genotype	Sample	Proportion of bisexual flowers	Remarks
1	DPC 9	Female flowers/ buds from terminal or subterminal	2/10<br>4/18<br>3/15	Bisexuality (rudimentary stamens) indicated reversion in female flowers.

		position (10 opened, 18 partially opened and 15 unopened)	Total: 9/43=20.93%	
2	DPC 17-S3	Flowers/buds appearing externally as female	4/4=100%	Flowers with rudimentary stigma (2 nos) and rudimentary stamens (2 nos were observed
3	M 574-OS1	Terminal flowers/buds of the spike collected at random.	6/14; 42.85%	Flowers with rudimentary stigma (1 no), with rudimentary tubular style and fused stigma (2 nos) and typical bisexual flowers with well-developed stigma (3 nos) were observed
4	DPC 9-OS2	Terminal flowers/buds of the spike collected at random	3/4= 75%	Flowers with rudimentary stamens (2 nos) and bud externally appearing as male but bisexual with ovary and stigma (1 no)
5	DPC 107	Terminal flower buds	Nil	All terminal buds female
6	DPC 21	Terminal flower buds	Nil	All terminal buds female

C. Bisexuality observed in inflorescences of castor genotypes (July 1st week, 2017)

**Genotype**	**Phenotype**	**Number of inflorescences analysed**	**Total number of flowers**	**Number of bisexual flowers**
RG-156	Monoecious	15	506	4 (0.79%)
DPC 17-S3	Apical male	18	833	12 (1.44%)
M574-OS1	Staminate (Terminal hermaphrodite)	27	561	1 (0.17%)
DPC 9-OS2	Staminate (Terminal hermaphrodite)	6	240	2 (0.83%)

D. Alteration of sexuality of terminal flower to female in staminate genotypes by July, 2017

**M574-OS1**			
**Inflorescence /Branch order**	**No. of bisexual flowers**	**No. of female flowers**	**Remarks**
4	0	0	All male
5	0	1	Tip female, Stage V of inflorescence
6	1	0	Tip bisexual rudi stamens
6	0	0	All male
6	0	0	All male

7	0	3	Tip male
7	1	0	Tip bisexual rudi stamens
7	0	0	All male
7	0	0	All male
8	0	7	Tip male, lower branch many female flowers
8	0	2	Tip female
8	0	1	Tip female
10	1	0	Tip bisexual
10	0	1	Tip female
**DPC 9-OS2**			
4	0	0	Tip male, Stage VII
4	0	1	Tip female, Rest male, Stage V
5	0	0	Tip male Stage VII,
5	0	1	Tip female, rest male
6	0	0	Tip male, Stage IV complete male
6	0	1	Tip female
6	0	4	Tip female, many female near tip
6	0	1	Tip female, rest male
7	0	1	Tip female, Stage VII
8	0	1	Tip female, Stage VII
8	0	1	Tip female

Supplementary Table S5. Weather parameters during sample collection in different castor genotypes used for scanning electron microscopy

CDD: Cumulative Degree Days; GDD: Growing Degree Days; T_{max}: Daily maximum temperature; T_{min}: Daily minimum temperature; T_{avg}: Daily average temperature. Mean of the values for 2 weeks before date of sample collection are given.

Date of Sample collection	Cropping Season	Days After Planting (DAP)	Weather parameters (2 weeks before sample collection)				
			CDD (from date of sowing)	Mean GDD ± SE (Mean)	Mean T_{max} ± SE (Mean)	Mean T_{min}± SE (Mean)	Mean T_{avg} ± SE (Mean)
**RG 156**							
19-09-2014	2014-2015	60	983.5	16.0 ± 0.45	29.3 ± 0.89	22.7 ± 0.17	26.0 ± 0.45
28-10-2014		99	1618.4	15.2 ± 0.53	31.2 ± 0.96	19.2 ± 0.13	25.2 ± 0.53
27-01-2015		190	2660.6	9.5 ± 0.38	28.5 ± 0.29	10.5 ± 0.61	19.5 ± 0.38
03-03-2015		225	3129.2	15.5 ± 0.59	33.4 ± 0.51	17.5 ± 1.01	25.4 ± 0.59
06-04-2016	2015-2016	262	4031.8	19.8 ± 0.26	38.1 ± 0.24	21.5 ± 0.45	29.8 ± 0.26
**DPC 21**							
26-01-2015	2014-2015	189	2650.1	9.4 ± 0.35	28.3 ± 0.29	10.5 ± 0.61	19.4 ± 0.35
02-02-2015		196	2725.3	10.4 ± 0.28	28.9 ± 0.23	12.0 ± 0.50	20.4 ± 0.28
13-02-2015		207	2855.8	11.5 ± 0.41	29.8 ± 0.33	13.3 ± 0.64	21.5 ± 0.41
**DPC 9**							
19-09-2014	2014-2015	60	983.5	16.0 ± 0.45	29.3 ± 0.89	22.7 ± 0.17	26.0 ± 0.45
28-10-2014		99	1618.4	15.2 ± 0.53	31.2 ± 0.96	19.2 ± 0.13	25.2 ± 0.53
27-01-2015		190	2660.6	9.5 ± 0.38	28.5 ± 0.29	10.5 ± 0.61	19.5 ± 0.38
13-02-2015		207	2855.8	11.5 ± 0.41	29.8 ± 0.33	13.3 ± 0.64	21.5 ± 0.41
25-06-2015		339	5372.1	17.8 ± 0.6	31.4 ± 0.84	24.2 ± 0.46	27.8 ± 0.6
27-11-2015	2015-2016	131	2083.0	13.5 ± 0.35	29.6 ± 0.51	17.4 ± 0.63	23.5 ±0.35
06-04-2016		262	4031.8	19.8 ± 0.26	38.1 ± 0.24	21.5 ± 0.45	29.8 ± 0.26
**M574-OS1**							
19-09-2014	2014-2015	60	983.5	16.0 ± 0.45	29.3 ± 0.89	22.7 ± 0.17	26.0 ± 0.45
28-10-2014		99	1618.4	15.2± 0.53	31.2 ± 0.96	19.2 ± 0.13	25.2 ± 0.53
13-06-2015		327	5167.5	22.0 ± 0.40	37.4 ± 0.71	26.5 ± 0.29	32.0 ± 0.40
26-11-2015	2015-2016	130	2070.5	13.6 ± 0.32	29.7 ± 0.52	17.4 ± 0.61	23.6 ± 0.32
06-04-2016		262	4031.8	19.8 ± 0.26	38.1 ± 0.24	21.5 ± 0.45	29.8 ± 0.26

Supplementary Table S7. Castor genotypes with distinct sex expression used for the study.

* M 574-OS1 (green stem and spiny capsules) and showing bisexual flowers at the tip (frequency 3/20) were found among monoecious red stem plants. The next generation segregated into green and red stem plants with spiny and non-spiny capsules respectively. The inflorescence of quaternary orders and above which were completely male were taken for studies.

Sl. No	Line	Stem colour	Bloom	Sex expression
1	DCS 107	Green	Double	Monoecious variety with 3-4 whorls or 30-50 % male flowers at bottom and female flowers on top. Female flowers also found in lower whorls
2	RG 156	Green	Single	Monoecious germplasm accession with 70-80% male flowers at bottom and female flowers on top> No female flowers seen in lower whorls
3	DPC 9	Green	Nil	Pistillate line, Late revertant
4	DPC 9 outcrossed selection-OS2	Green	No bloom/ single bloom in segregants	Pistillate line, has bisexual flowers in tip and near tip in lower orders and monoecious with male flowers in lower orders of branching. Completely male inflorescence in higher order above 4th branching order. Used as male line in the study
5	DPC-16	Red	Nil	Pistillate line, tip flower bisexual, behaves as monoecious with some branch orders having male flowers. 5 different phenotypes observed. viz., Completely pistillate, Pistillate with terminal bisexual flower and few bisexual flowers, pistillate with terminal male flower, Predominantly pistillate with few random male flowers and male or female terminal flower
6	DPC-17-Selection S3	Red	Double	Apical Interspersed male flowers (ISF)
7	DPC-21	Green	Double	Pistillate line with ISF (Interspersed staminate flowers) seen throughout inflorescence after capsule setting.
8	*M574-OS1 outcrossed selection-OS1	Green	Double	Monoecious variety with less number of female flowers and terminal bisexual till 3rd order of branching. Above 3-4th branch order fully male inflorescence. Used as male line

Supplementary Table S8. Number of inflorescences used for studies on stage transitions, growth and architecture of inflorescence in various castor genotypes.

A. Number of buds tagged for studies on stage transition of castor inflorescence

Sl No	Variety	Stage 1	Stage 2	Stage 3	Stage 4				Total
					A	B	C	Total	
1	DCS 107	10	5	4	2	2	1	5	24
2	RG 156	-	5	9	5	-	-	5	19
3	DPC 9	4	3	4	5	2	-	7	18
4	DPC 21	2	15	4	2	-	-	2	23
5	DPC 17 S3	-	14	4	-	-	6	6	24

B. Number of inflorescences used for floral architecture studies

Sl No	Variety	Branch order			Total
		Secondary	Tertiary	Quaternary	
1	DCS 107	-	13	-	13
2	RG 156	6	1	14	21
3	DPC 9	-	12	10	22
4	DPC 17 S3	1	17	-	18
5	M574-OS1	-	-	8	8

Supplementary Table S9. Sequence of primers or oligonucleotides used for amplification of control and candidate genes.

Sl. No	Gene	Gene ID	Forward Primer 5´- 3´	Reverse Primer 5´- 3´	**Product size** using RNA/RT-PCR (bp)	**Product size** using genomic DNA (bp)
**Control genes**						
1	EF-1<br>(Elongation Factor -1 Delta)	8266315	AGGAGTATTCAGATGGAAGGAC	TTGAAGGCCACAATATCACAAC	191	482
2	UBQ<br>(Ubiquitin-40S ribosomal protein S27a)	8265906	GCGGAAAGATGCAGATCTTCG	TCCTCTCAATCGCAGCACCAG	233	233
3	ACT<br>(Actin)	8267071	TTCCCAGGCATTGCTGATAG	TGTGGACAATTGATGGTCCAG	197	531
4	PGK<br>(Phosphoglycerate Kinase)	8259993	TGGACCTGATGCGATCAAGAC	ACACCCTTTCCACTGAGCTC	153	235
5	GADPH<br>(Glyceraldehyde-3-phosphate dehydogenase, Cytosolic)	8272110	TACACTGATGAAGATGTCGTC	ACAGTCTTTCTCATTGCACAG	193	488
6	SND<br>(Sand protein homologue/ Vacuolar fusion protein MON1 homologue)	8261010	CATATGTTGGCATTGGTGGTC	TGAGTCTTGTGTGGACCATTC	190	304
**Candidate genes**						
1	ACS<br>(1-aminocyclopropane-1-carboxylate synthase)	8287433	GCATCGTTATGAGCGGAGG	TGGGATCAGTTGAACTCCTG	149	1096
2	ACS-1<br>(1-aminocyclopropane-1-carboxylate synthase 1)	8286682	CAATCGGGTAACGTTCGATC	AATCTTCACACCAGTTCGCC	169	1131
3	DEF<br>(Deficiens)	8273018	GAGTTCATTAGCCCTGGCAC	TCAGATCCTCACCCATCCTC	175	419
4	SDR<br>(Short-chain dehydrogenase reductase 2a)	8287923	GCCAGAGCAAACCCTTCATG	GGCTAGTATTGTTCCAGGAG	199	479

5	WUS<br>(Wuschel)	8267096	GCATCTTCATCTTCAACTACTG	TTGATCTTCTCCATCTTCTAGG	219	718
